# Antibacterial Properties, Arabinogalactan Proteins, and Bioactivities of New Zealand Honey

**DOI:** 10.3390/antiox14040375

**Published:** 2025-03-21

**Authors:** Emey M. George, Swapna Gannabathula, Rushan Lakshitha, Ye Liu, Kevin Kantono, Nazimah Hamid

**Affiliations:** Department of Food Science, Auckland University of Technology, Private Bag 92006, Auckland 1142, New Zealand; emeyg92@yahoo.com.au (E.M.G.); swapna.gannabathula@aut.ac.nz (S.G.); ycn4788@autuni.ac.nz (R.L.); ye.liu@aut.ac.nz (Y.L.); kkantono@aut.ac.nz (K.K.)

**Keywords:** honey, antibacterial, arabinogalactan proteins, AGPs, MGO, New Zealand honey kānuka honey, kāmahi honey, pōhutukawa honey, mānuka honey, rewarewa honey, clover honey, thyme honey, honeydew honey, polyphenols, flavonoids, vitamin C, bioactivities, health, growth inhibition assay, radial gel diffusion assay, ferric reducing antioxidant power assay, FRAP, cupric ion reducing antioxidant capacity assay, CUPRAC, radical scavenging capacity, DPPH, Folin–Ciocalteau total phenolic content assay, TPC, liquid chromatography-mass spectrometry, LCMS

## Abstract

Honey has been used for centuries for its antibacterial and healing properties. The aim of this study was to investigate the antibacterial properties, arabinogalactan proteins (AGPs), antioxidant activities, and polyphenolic content of eight different types of New Zealand honey (clover, mānuka, beech honeydew, pōhutukawa, kānuka, rewarewa, kāmahi and thyme honey). The results showed varying antibacterial activities across the honey types, with mānuka, pōhutukawa, and kāmahi honey exhibiting significant inhibitory effects. Interestingly, all honey samples demonstrated inhibitory effects on bacterial growth at 25% concentration. Furthermore, AGPs were found in all eight honey samples, with higher amounts in kānuka, kāmahi, pōhutukawa, mānuka, and rewarewa honey. Thyme had the highest antioxidant values in terms of CUPRAC, FRAP and DPPH, while kāmahi honey had the lowest antioxidant value. Beech honeydew honey had the highest Total Flavonoid Content (TFC) values, while thyme and clover honey had the lowest TFC values. Similarly, thyme honey exhibited the highest Total Phenolic Content (TPC) value, with kāmahi and clover honey having the lowest TPC values. Furthermore, only thyme and beech honeydew New Zealand honeys contained vitamin C. The different honeys contained varying concentrations of polyphenols, with mānuka, kānuka, and pōhutukawa honeys having high amounts of quercetin, luteolin, and gallic acid, respectively. In contrast, clover honey had notable levels of chrysin, pinocembrin, caffeic acid, and pinobanksin. Overall, this study provides valuable insights into the antibacterial properties and bioactivities of native New Zealand honeys, contributing to our understanding of the potential health benefits associated with these honeys and their potential use as natural alternatives to improve human health.

## 1. Introduction

Honey is recognised for its antibacterial effects, known to effectively inhibit the growth of bacteria in wounds [1]. These potent antibacterial properties stem from a combination of mechanisms [2]. Firstly, the high sugar content creates a hypertonic environment that dehydrates and kills bacterial cells by drawing out water. Additionally, the natural acidity of honey, ranging from 3.2 to 4.5, hinders the growth of many bacteria. When honey is diluted, the enzyme glucose oxidase is activated, leading to the production of hydrogen peroxide, a powerful antibacterial agent. Honey also contains polyphenols and phytochemicals with antimicrobial properties that can harm bacterial cell walls and membranes. Additionally, Al-Sayaghi et al. [3] reported that high concentrations of MGO in mānuka honey can disrupt bacterial cell functions by interfering with proteins and DNA. Mānuka honey is also capable of inhibiting bacterial protein synthesis, preventing bacterial growth and reproduction. The primary antibacterial effects of honey are attributed to the presence of hydrogen peroxide that is produced when glucose oxidase breaks down glucose into gluconic acid and hydrogen peroxide [4,5,6]. Although the exact mechanism by which honey inhibits the growth of bacteria is not fully understood, research suggests that it could involve oxidative stress induced by hydrogen peroxide. However, this would require a significant amount of hydrogen peroxide to produce the desired effect. Additionally, it is proposed that the interaction between phenolic acids and hydrogen peroxide leads to bacterial growth inhibition and the disruption of DNA strands in bacteria [7].

Catalase, which is abundant in open wounds, can break down hydrogen peroxide, potentially decreasing the antibacterial effect of honey when used in wound care [5]. Mānuka honey contains methylglyoxal (MGO), which behaves in a similar manner to hydrogen peroxide. MGO is formed when a precursor dihydroxyacetone (DHA) is non-enzymatically converted into MGO [8,9]. There is some form of synergism between MGO and other compounds present in mānuka honey that is responsible for its high antibacterial effect [10]. It is important to note that while studies have been conducted on the antibacterial effects of various native honeys produced in New Zealand, none have exhibited the same level of antibacterial activity as mānuka honey [4,11].

Arabinogalactan proteins (AGPs) are proteoglycans that are present on the cell surface of plants and their exudates, which play a critical role in plant defence mechanisms [12]. Initially, the immunostimulatory properties of honey were attributed to lipopolysaccharides (LPS), which are endotoxins found in the cell walls of Gram-negative bacteria [13]. However, Gannabathula, Krissansen [14] debunked this theory and found that it was not the amount of LPS present in honey that stimulated the production of Tumour Necrosis Factor-alpha (TNF-α). TNF-α is a cytokine that plays a major role in inflammatory responses and is involved in the pathogenesis of inflammatory and autoimmune diseases. They found that it was type II AGPs that stimulated TNF-α. Furthermore, the authors found that pure AGPs extracted from honey produced very low amounts of TNF-α on their own, and that apisimin—a peptide secreted from the glands of honeybees into Royal Jelly—also had the ability to stimulate TNF-α production in low amounts. Interestingly, when both AGPs and apisimin were present, high levels of TNF-α were produced. It was postulated that both molecules worked synergistically to produce immunostimulatory activity in honey, with kānuka honey containing more AGPs compared to mānuka and clover honey [14]. However, no further studies have yet investigated the presence of AGPs in other New Zealand native honeys.

Phenolic compounds are the main contributors to the antioxidant properties of honey. However, other elements such as enzymes, amino acids, and carotenoids potentially have a role [15]. Several studies have investigated the antioxidant capacity of New Zealand honey (a large portion are particularly focused on just Mānuka honey [15,16,17,18,19,20,21,22,23]) but only a few have examined other types of New Zealand honeys. Phenolic compounds, including flavonoids, phenolic acids, lignans, and stilbenes, are known for their antioxidant characteristics and for additional antibacterial, antiviral, and anti-inflammatory properties [24,25]. The phenolic composition and antioxidant activities of honey are influenced by factors like floral origins, seasonal and climatic variations, and processing methods [19,20,21,22,23,26]. The major polyphenols found in honey are flavonoids, phenolic acids, and phenolic acid derivatives. Flavonoids, a type of polyphenolic compound, are the main class of secondary metabolites found in honey and are responsible for many of its biological effects [27,28]. The most common flavonoids found in mānuka honey are luteolin, pinobanksin, quercetin, kaempferol, pinocembrin, and rutin [19,20,21,23]. However, research specifically on the total flavonoid content in New Zealand honey is limited, with existing studies primarily concentrating on the presence of flavonoids in Mānuka honey [17,20,29,30].

Vitamin C, an essential nutrient for humans with antioxidant properties and crucial roles in collagen synthesis, wound healing, and immune function [31,32], may also serve as a marker for differentiating honey derived from specific botanical sources. However, studies solely focused on determining the vitamin C content of New Zealand honey are lacking. Nevertheless, research conducted on European thyme honey, which shares the same botanical species as New Zealand thyme (brought to NZ during the late-1800s gold rush), has shown high levels of vitamin C [33,34,35]. Vitamin C is sensitive to high temperature, air, and light, leading to its degradation in commercial honey [36].

There have been relatively few studies conducted on the antibacterial effects of New Zealand honey, except for mānuka honey [4,11,37]. Only one study has investigated the presence of AGPs in honey [38], and few studies have examined the polyphenols present in New Zealand honeys, primarily focusing on mānuka honey [19,20,21,22,23]. Overall, this indicates the need for more comprehensive research to explore the polyphenolic content, antibacterial effects, and presence of AGPs in New Zealand native honeys beyond mānuka honey. Therefore, the aim of this research was to determine the antibacterial effects, AGP content, and polyphenolic constituents in eight New Zealand native honeys. This will provide an understanding of the unique properties and potential health benefits of different New Zealand native honeys.

## 2. Materials and Methods

### 2.1. Honey Samples

The experiments were carried out with eight honey samples originating from New Zealand collected between the years 2018 to 2020. The honey samples were defined and classified based on Standard 2.8.2 by the Australia New Zealand Food Standards Code. The honeys were grouped based on the dominant pollen or most common pollen from a high density of plants of a particular floral variety. The samples included clover (*Trifolium repens*), mānuka (*Leptospermum scoparium*), beech honeydew (*Nothofagus solandri*), Pōhutukawa (*Metrosideros excelsa*), kānuka (*Kunzea ericoides*), rewarewa (*Knightia excelsa*), kāmahi (*Weinmannia racemosa*), and thyme (*Thymus vulgaris*). The honey samples were stored under controlled conditions to ensure the preservation of their properties and prevent degradation or alteration. Each sample was stored in a dark, airtight container at 4 °C until analysis. These specific floral varieties were chosen for their prevalence in New Zealand and their importance in the honey industry. Known for their unique properties and potential health benefits, each variety holds value for both commercial and research applications.

**Figure 1:**
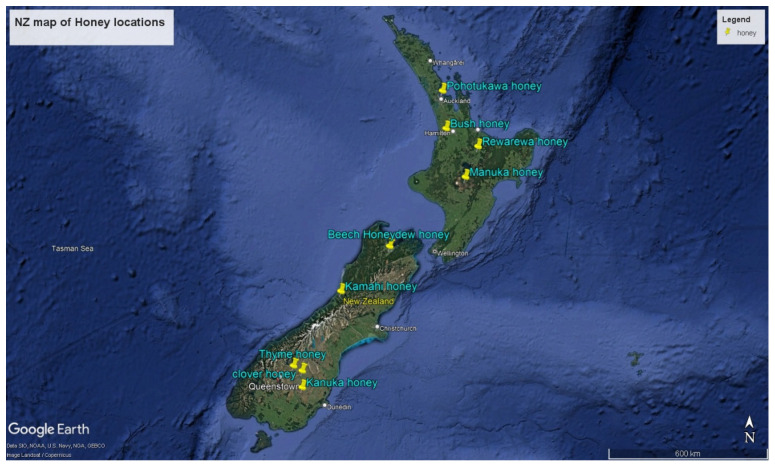
Map of New Zealand indicating the locations of honey sample collection. Source: Google Earth Pro accessed October 2024.

The honey samples were collected from different locations across New Zealand, as illustrated in Figure 1. These locations represent different regions with distinct climatic and geographical features, influencing the distinctive flavours and properties of the honey produced.

### 2.2. Broth Assay

#### 2.2.1. Bacterial Strains and Media

Eight different strains of bacteria were used to determine the antibacterial effects of honey samples. The Gram-positive bacteria included *Staphylococcus aureus* ATCC 25923, *Staphylococcus epidermidis* ATCC 12228, *Bacillus subtilis* (AUT lab culture), *Enterococcus faecalis* ATCC 29212 and *Lactobacillus plantarum* (AUT lab culture). Gram-negative bacteria included *Salmonella typhimurium* (AUT lab culture), *Escherichia coli* ATCC 25922, and *Pseudomonas aeruginosa* ATCC 27853.

Most cultures used were grown using Tryptic Soy Broth (TSB) (BD Difco, Plymouth, UK), except for *L. plantarum*, which was grown on de Man, Rogosa and Sharpe broth (MRS) (Thermofisher Scientific, Waltham, MA, USA). *S. aureus* was grown on TSB broth with the addition of 10% NaCl and 1% sodium pyruvate, for optimal growth. All bacteria were sub-cultured on either Mannitol Salt Agar plates (BD Difco, Plymouth, UK) or Nutrient Agar plates (BD Difco, Plymouth, UK), except for *L. plantarum*, which was grown on MRS Agar plates (Thermofisher Scientific, Waltham, MA, USA).

#### 2.2.2. Bacterial Culture

Single colonies were sub-cultured from cultures obtained from the Food Science and Microbiology Department, Auckland University of Technology (AUT), onto appropriate agar plates and incubated overnight at 35 °C in temperature-controlled rooms, Auckland, New Zealand. The plates were then stored in the refrigerator until required for the experiment.

Single colonies from the plates were inoculated into the appropriate broths and incubated overnight at 35 °C. To ensure that the bacterial colonies were not in the stationary phase (lag phase) for too long, which would delay their exponential growth (log phase), 10 μL of this broth was further inoculated into another tube of broth and incubated overnight. The cell density of these overnight cultures was approximately 10^9^ colony-forming units (CFU)/mL. This culture was then diluted until the cell density was approximately 10^3^ CFU/mL. Cell density was measured using a spectrophotometer (Pharmacia Biotech Ultrospec 2000, Stockholm, Sweden) at an optical density of 600 nm. The culture was diluted using double-strength broth, which was made with twice the amount of broth powder. This double-strength broth would be further diluted by half when added to the honey solution, thereby becoming a single-strength broth solution.

### 2.3. Growth Inhibition Assay

Growth inhibition assay was carried out following the method described by [37]. A 50% (*w*/*v*) honey solution was freshly prepared by weighing the appropriate amount of honey in an Eppendorf tube and adding a specified volume of sterile water. The mixture was vortexed for approximately 2 min and then stored at 35 °C for 30 min to completely dissolve any remaining sugar crystals. Next, 100 μL honey solution was then placed in rows 1 and 2 of a 96-well microtitre plate. An equal volume (100 μL) of sterile water was then added into row 2, and the solution was aspirated to ensure even dilution. Then, 100 μL of this dilution was transferred into the next row to further dilute the honey solution by half. This dilution step was repeated to achieve final concentrations of honey solutions at 25%, 12.5%, 6.25% and 3.125%.

The bacterial inoculum was added to each well, bringing the final volume to 200 μL and diluting the honey concentration further by half. To investigate the effects of high sugar content present in honey on bacterial growth, an artificial honey solution was prepared using 192 mg fructose, 180 mg glucose, 4 mg sucrose and 10 mL sterile water. This artificial honey solution was diluted and used in the same manner as the honey solutions. For control purposes, the positive control consisted of the bacterial culture alone, while the negative control involved using the broth media itself.

The 96-well microtiter plate was placed in a microtitre plate reader (FLUOstar Omega version 5.0, BMG Labtech, Ortenberg, Germany) set at an optical density of 600 nm to measure the cell growth in terms of turbidity over 48 h at 35 °C. The machine was automatically set with moderate shaking at 500 rpm and double orbital shaking. Four technical replicates were conducted for each honey sample concentration and bacteria. Results were measured using optical density (OD), and the bacterial growth curves were generated using the average of all four replicates. ANCOVA (Analysis of Covariance) was used to analyse the bacterial growth curve data from a broth microdilution assay with honey. ANCOVA is widely used to compare the slopes of regression functions, such as the growth curves of individuals or populations. In this context, the focus is on comparing the effects of different factors (e.g., honey concentration) on the dependent variable (OD) while controlling covariates (in this case, time). In this analysis, OD measurements at each time point were used as the dependent variable, and time was included as a covariate to control for its effect. This approach allows us to determine whether honey concentration significantly affects bacterial growth while accounting for changes over time. ANCOVA provides a more detailed analysis by considering these time-dependent changes. Additional checks indicated that all responses were highly significant (*p* < 0.001). Fisher’s post-hoc test was also performed to further elucidate the differences in bacterial concentration for each honey sample.

The broth microdilution assay was the best choice for conducting this test, as it was sensitive enough to detect small changes. Methods such as agar well-diffusion assays were not as sensitive, and honey samples could have been further diluted by seeping through the agar—they are better suited for studying wound site behaviour of honey [4]. Although the agar well-diffusion method was used as a preliminary method in this study, the broth dilution method was employed instead, as most of the diffusion assay results were not detectable. Hence, a highly sensitive method, such as broth microdilution, was used to identify inhibition even at the lowest honey concentration.

### 2.4. Radial Gel Diffusion Assay

The radial gel diffusion method described by Van Holst and Clarke [39] was used to quantify the amount of AGPs present in the honey samples, with few modifications. The amount of AGPs was quantified using a known quantity of gum Arabic (standard) for reference. A mixture of 15 μM of β-Glucosyl Yariv reagent (GlycoSyn, Glycofinechem, Lower Hutt, New Zealand) was prepared with 1% agarose (Sigma Aldrich, Bayswater, VIC, Australia) containing 0.15 M NaCl and 0.02% sodium azide. This gel (20 mL) was poured onto the plates and allowed to set. Wells with a diameter of 5 mm diameter were punched in the agar.

The honey samples (30 µL) were diluted 1:1 in a buffer containing 0.15 M NaCl and 0.02% sodium azide, and the diluted samples were added into the wells. The buffer was used as a negative control, while gum Arabic (Sigma Aldrich, Bayswater, VIC, Australia) served as a positive control. The plate was sealed and incubated in the dark at room temperature for 24–48 h until an orange-coloured precipitin halo developed around the wells. To aid in observation, a 1% agarose gel was poured on top of the gel to enhance the visibility of the halo precipitation around the wells. A picture was taken to calculate the area and measure the concentration of AGPs using Image J (1.50d) (LOCI, University of Wisconsin, Madison, WI, USA). All samples were tested in triplicates, and the halo precipitation of the samples was compared to the halo precipitation produced by the standard, with the area of precipitation being calculated.

### 2.5. Ferric Reducing Antioxidant Power Assay (FRAP)

The FRAP assay was analysed using the method described by [18]. All types of honey were dissolved in distilled water to obtain a concentration of 0.05 g/mL. For the FRAP assay, acetate buffer (0.3 M, pH 3.6), HCl solution (0.04 M), TPTZ reagent (0.01 M in 0.04 M HCl), and FeCl_3_ solution were prepared. FeCl_3_ and TPTZ solutions were prepared fresh daily before the assay.

FRAP reagent was prepared by adding 1 mL of TPTZ and FeCl_3_ with 10 mL of acetate buffer. After mixing, the reagent was heated to 36 °C. Aliquots of 100 µL of honey solution were mixed with 2 mL of FRAP reagent and 900 µL of H_2_O. The absorbance of the reaction mixture was measured using a UV spectrophotometer at 593 nm after vortexing and leaving it aside for 4 min. Trolox (5–80 ppm) was used for the calibration curves, and the results were expressed as mg of Trolox equivalents per kg of honey.

### 2.6. Cupric Ion Reducing Antioxidant Capacity (CUPRAC) Assay

The CUPRAC assay was performed following the protocol described by [18]. All types of honey were dissolved in distilled water to achieve a concentration of 0.05 g/mL. For the CUPRAC assay, ammonium acetate buffer (1 M, pH 7), CuCl2 solution (0.01 M), and neocuproine solution (0.0075 M in ethanol) were prepared.

A series of Trolox solutions with concentrations ranging from 10 ppm to 80 ppm were prepared. For each Trolox solution, 1 mL of Trolox was mixed with 1 mL of each of the three previously prepared solutions and 0.1 mL of deionised water. The mixture was allowed to react for 5 min and then the absorbance was measured at 450 nm against a blank. Trolox (10–80 ppm) was used to create calibration curves, and the results were expressed as mg of Trolox equivalents per kg of honey.

### 2.7. Determination of Radical Scavenging Capacity Against DPPH

The DPPH assay was analysed using the method described by [36], with minor modifications. All honey samples were dissolved in distilled water to achieve a concentration of 0.20 g/mL. A solution of 250 mL buffered ethanol was prepared by mixing 100 mL of the 0.1 M acetate buffer with 150 mL of absolute ethanol. To prepare the 0.2 mM 2,2-diphenyl-1-picrylhydrazyl (DPPH) solution, 0.0079 g of DPPH was dissolved in 100 mL of methanol.

In a 15 mL Falcon tube, a control solution was prepared by mixing 0.50 mL of buffered ethanol, 2 mL of DPPH solution, and 4 mL of methanol. For the blank solution, 0.50 mL of buffered ethanol was added to 6 mL of methanol. The blank solution served as the reference.

The dissolved honey sample (0.50 mL) was mixed with 2 mL of the DPPH solution and 4 mL of methanol. The mixture was then stored in a dark place at room temperature for 30 min, and the absorbance was measured at 517 nm. A series of Trolox solutions were prepared and used to create a calibration curve. The results were expressed as mg of Trolox equivalents per kg of honey. The calculation of DPPH inhibition is shown in the equation below:

$$Inhibition\;of\;DPPH\left(\%\right)=\left(\frac{A_{c}-A_{s}}{A_{c}}\right)\times 100$$

A_c_: absorbance of control.

A_s_: absorbance of sample.

### 2.8. Folin–Ciocalteau Total Phenolic Content (TPC) Assay

The total phenolic content of honey samples was determined using a colourimetric assay using the Folin–Ciocalteau phenol reagent [18]. Specifically, a 1 mL aliquot of a diluted honey sample (0.125 g/10 mL water) was mixed with 0.5 mL of Folin–Ciocalteau phenol at room temperature for 5 min. Next, a 1.5 mL solution of 10% (*w*/*v*) Na_2_CO_3_ was added to the mixture, which was then incubated for 2 h in a dark place. Following the incubation period, the absorbance of the solution was measured using a spectrophotometer set at a wavelength of 765 nm. The total phenolic content was quantified as milligrams of gallic acid equivalents (GAE) per kg of honey.

### 2.9. Total Flavonoid Content

The total flavonoid content in honey was determined using the aluminium chloride colorimetric method following the procedure outlined by [40], with slight modifications. Honey was dissolved in distilled water to obtain a concentration of 0.25 g/mL. The honey sample (1.5 mL) was mixed with 75 µL of 5% (*w*/*v*) NaNO_2_ solution and incubated at room temperature for 6 min. Then, 150 µL of 10% AlCl_3_ solution was added and incubated at room temperature for another 6 min. Finally, with the addition of 0.5 mL of 1 M NaOH and the total volume made up to 2.5 mL with deionised water, the mixture was then incubated at room temperature for 15 min until the colour turned pink. The absorbance was measured at 510 nm using a spectrophotometer. The results are expressed as rutin equivalents (REs) (mg RE/g honey extract).

### 2.10. Vitamin C Analysis

The vitamin C content of honey was determined using the procedure outlined by [33] with slight modifications. Each honey sample (10 g) was completely dissolved in 10 mL 0.25% meta-phosphoric acid (MPA) in distilled deionised water. The solution was filtered through a membrane filter (0.45 μm), and aliquots (10 μL) of the filtrate were injected into the HPLC for the determination of ascorbic acid. For total vitamin C quantification, 300 μL of honey solution (1 g/1 mL) was added to 30 μL of dithiothreitol solution (20 mg/mL), and the mixture was kept in the dark at room temperature for about 2 h. After that, 300 μL of 0.05% MPA was added, and the mixture was vortexed for 30 s. Finally, the mixture was filtered through a 0.45 μm Millipore membrane and injected into the HPLC system. The dehydroascorbic acid (DHAA) was calculated as the difference between the total vitamin C (after reduction) and ascorbic acid (AA).

The analysis was performed using a high-performance liquid chromatograph equipped with an ultraviolet detector (Shimadzu Corp., Kyoto, Japan). Chromatographic separation was carried out with a Synergy polar-RP C18 (250 mm × 4.6 mm i.d, 5 μm particles) column. The mobile phase was MeOH: H_2_O (15:85), pH = 2.5, adjusted with MPA at a flow rate of 1 mL/min, and the detection was carried out at 254 nm. All measurements were carried out at room temperature.

### 2.11. Polyphenol Analysis Using Liquid Chromatography-Mass Spectrometry

The honey sample extracts for polyphenol analysis were prepared according to the method outlined by Sun, Tan [41]. Honey samples (2 mg) were mixed with 8 mL of Milli-Q water (Milli-pore, Bedford, MA, USA), and the pH was adjusted to 7 using 5% ammonium solution (Fisher Chemicals, Fair Lawn, NJ, USA). The mixture was then centrifuged at 4000 rpm for 20 min to remove solid particles.

Phenomenex Strata XA cartridges (Phenomenex, Auckland, New Zealand) were used to isolate polyphenols. The cartridges were conditioned with 3 mL of methanol (Fisher Chemicals, Fair Lawn, NJ, USA), followed by 3 mL of Milli-Q water at pH = 7. The supernatant from the centrifugation step was loaded into the cartridge, followed by 4 mL of Milli-Q water at pH = 7. The phenolic compounds retained in the cartridge were eluted using 5 mL of methanol/formic acid (1:9 *v*/*v*) (Fisher Chemicals, Fair Lawn, NJ, USA). The eluate was then evaporated under a fume hood using an evaporator. The eluate was dried to approximately 0.75 mL, and reconstituted with 2 mL of methanol, and mixed with 2% acetic acid (Fisher Chemicals, Fair Lawn, NJ, USA). Prior to LC-MS analysis, all the samples were filtered using a 0.22 nm filter (Milli-pore, Bedford, MA, USA).

Commercial standards of polyphenols (rutin, quercetin, kaempferol, luteolin, gallic acid, benzoic acid, caffeic acid, p-coumaric, pinobanksin, chrysin, epicatechin, catechin, apigenin-7-o-glucoside, pinocembrin, hydroxybenzoic acid, homovanillic acid and quinic acid), all acquired from Sigma Aldrich (Castle Hill, NSW, Australia), were used to determine the concentration of polyphenols present in the honey samples.

LC-MS analysis was conducted using an Agilent 1260 Infinity Quaternary LC System (Agilent, Santa Clara, CA, USA) connected to a 6420 triple quadrupole LC/MS system with an electrospray ionisation (ESI) source (model number: G1948B). The system components included a 1260 quaternary pump (model number: G1311B), a 1260 infinity ALS sampler (model number: G1329B), a 1260 infinity TCC column component (model number: G1316A), and a 1260 infinity diode array detector (DAD) (model number: G4212B). 

The MS ionisation source conditions were as follows: a capillary voltage of 4 kV, a drying gas temperature of 300 °C, a drying gas flow of 10 L/min, and a nebuliser pressure of 40 psi. Quantitative analysis in the negative ionisation mode was performed using multiple reaction monitoring (MRM). The MRM transitions with collision energy and cell accelerator voltage are summarised in Supplementary Table S1, which also provides the retention times and chemical formulas of the different polyphenolic compounds.

The Agilent Poroshell EC-C18 (2.1 × 150 mm, 2.7 µm) column was used. The mobile phases consisted of water containing 0.1% (*v*/*v*) formic acid (A) and acetonitrile containing 0.1% (*v*/*v*) formic acid (B). The flow rate was set at 0.30 mL/min, and the column temperature was maintained at 40 °C. The initial gradient condition was 95:5 (A:B) and held for 5 min. From 5 to 8 min, the proportion of B was increased to 10% and held for 5 min. Betwen 13 and 16 min, B was increased to 30%, then to 45% from 16 to 18 min, where it was held for 5 min. From 23 to 25 min, B was increased to 80% and held for 3 min. From 28 to 29 min, B was decreased to 5%. The total run time was 40 min, and the injection volume was 5 µL.

### 2.12. Data Analysis

The AGP and polyphenol results were subjected to Analysis of Variance (ANOVA). For the broth assay results, ANOVA was independently performed for each honey concentration across all bacteria. For the antioxidant activity, vitamin C, and polyphenol results, ANOVA was performed, with sample repetitions included as a covariate. Fisher’s LSD post-hoc comparisons were carried out when statistical significance reached a 5% level.

In addition to ANOVA, partial least squares discriminant analysis (PLS-DA) was used to model the relationship between the honey samples and the polyphenols, antioxidant activity, and vitamin C content. In the PLS-DA model, the angle between the sample vectors and the polyphenol vectors indicates the strength of the correlation; a more acute angle indicates a stronger positive correlation, while a more obtuse angle indicates a weaker correlation. Additionally, the length of the vector arrows indicates the level of positive correlation, with longer arrows representing stronger correlations. Jack-knife cross-validation was utilised to visualise the dataset and identify key polyphenol fingerprints of the honey samples. All univariate and multivariate data analyses were carried out using the XLSTAT software version 2020.5 (Addinsoft, New York, NY, USA).

## 3. Results and Discussion

### 3.1. Antibacterial Effects of Honey

The broth dilution method was used to determine the susceptibility of microorganisms to various honey samples (% *w*/*v*). In the context of ANCOVA, the values in Table 1 represent the Least Squares Means (LSMeans), which are the adjusted means of the dependent variable (OD measurements) for each level of the independent variable (honey concentration), after accounting for the covariate (time). LSMeans provide an estimate of the mean OD for each treatment group (e.g., with and without honey) while accounting for the effects of other variables included in the model. This adjustment helps to isolate the effect of the treatment (honey) on OD, providing a clearer comparison between groups by controlling other influencing factors.

The results of this study indicate that all honey samples tested had a significant effect on the growth of bacteria. This significant effect refers to results obtained via the broth microdilution assay, which was sensitive enough to detect small changes. The effects of honey on bacterial growth varied depending on the type of honey and the concentration used. Mānuka honey demonstrated strong antibacterial effects by significantly inhibiting the growth of *E. coli* (F: 128.062, *p* < 0.0001), *E. faecalis* (F: 80.305, *p* < 0.0001), *L. plantarum* (F: 65.891, *p* < 0.0001), and *S. epidermidis* (F: 58.120, *p* < 0.0001), as seen in Table 1. Lu, Carter [37] found that mānuka honey had been reported to have the highest inhibition power, followed by the mānuka–kānuka blend, kānuka honey, and clover honey. The strong antibacterial effects in this study can be attributed to the MGO concentration of the mānuka honey being 850+ (equivalent to UMF 20+), as determined by the supplier [35]. MGO concentrations reported by Lu, Carter [37] were used for comparison. The authors used mānuka honey with MGO concentrations ranging between 651 and 1541 mg/kg (UMF 15+ to UMF 26+), while the mānuka–kānuka blends had MGO levels below 307 mg/kg of MGO (UMF 10+). Most of the mānuka honey sold in NZ falls within UMF 5+ (MGO: 83–219), 10+ (MGO 263–457) and 15+ (514–759). However, Molan [10] stated that high MGO levels do not necessarily result in high antibacterial activity. The author added that MGO must work synergistically with other compounds in the honey to enhance its antibacterial effects. Brady, Molan and Bang [11] found that mānuka honey had the highest antibacterial activity (28.4%) against *S. aureus* and a minimum inhibitory concentration (MIC) (the minimum required concentration to inhibit the growth of bacteria) of 6.3% with *E. coli*. In the current research, *E. coli* had one of the lowest MICs among all honey samples (not limited to mānuka honey) and was the most susceptible bacteria.

Kānuka honey was one of the most effective honey samples in inhibiting the growth of *S. aureus*, as observed in Table 1, consistent with the findings of Brady, Molan and Bang [11]. In their study, kānuka honey was found to inhibit the growth of *S. aureus* at an MIC of 3.125%, which contrasts the findings of Lu, Carter [37] who observed that kānuka honey was the least effective against *S. aureus* [35]. The authors also reported complete inhibition at high concentrations ranging between 16 and 32%. However, in the current study, some inhibition occurred at lower concentrations, with significant inhibition occurring between 12.5 and 25% concentration.

Pōhutukawa honey had a significant effect on *S. typhimurium* (F: 230.030, *p* < 0.0001), *P. aeruginosa* (F: 74.594, *p* < 0.0001)*, S. aureus* (F: 57.947, *p* < 0.0001), and *L. plantarum* (F: 31.747, *p* < 0.0001), as seen in Table 1. However, Brady, Molan and Bang [11] found that pōhutukawa honey had one of the lowest antibacterial activities and performed poorly with both *S. aureus* and *E. coli* among the 27 honey samples they tested. In the present research, pōhutukawa honey performed significantly better than other honey samples, displaying an MIC of 6.125% for both *S. aureus* and *E. coli*.

Pōhutukawa, kāmahi honey, and rewarewa honey significantly inhibited *S. typhimurium* even at the lowest concentration, as observed in Table 1. *S. typhimurium* showed high susceptibility to most honeys at 3.125% concentration, with thyme being the only sample that worked at 25% concentration. Artificial honey had no effect even at 25%. These findings suggest that high sugar levels did not impact *S. typhimurium’s* growth, and that other components present in honey were responsible for the observed inhibition at 3.125%. Our results align with the study by Mohapatra, Thakur and Brar [42], which also found that *S. typhimurium* was highly susceptible to honey, even more than standard antibiotics.

Artificial honey, followed by clover honey, had the least significant effect on the growth of the bacteria compared to other honey samples (Figures S8 and S1, respectively). Bacteria, such as *B. subtilis*, *E. faecalis*, *P. aeruginosa*, *S. epidermidis*, *S. aureus* and *L. plantarum*, were inhibited at 25% concentration of artificial honey. Interestingly, no effect on the growth of *S. typhimurium* was reported at this concentration in artificial honey. Clover honey did have some significant effect on bacterial growth, although it was less pronounced compared to other honey samples. Brady, Molan and Bang [11] reported that clover honey had one of the lowest antibacterial activities, with only 10.1% antibacterial activity against *S. aureus* and an MIC of 23.4% with *E. coli*. In our study, the MIC of clover honey required to inhibit the growth of *E. coli* was 3.125% concentration, whereas the MIC for *S. aureus* was 12.5% higher than most of the other honey samples that inhibited *S. aureus* at lower concentrations.

Only the highest concentration of honey (25%) inhibited *P. aeruginosa* for all eight honey samples, with rewarewa honey showing the most significant inhibitory effect at 25%, followed by pōhutukawa and kāmahi honey. Lu, Carter [37] also found that *P. aeruginosa* was inhibited at high concentrations of sugar between 16 and 32%. Rabie, Serem [43] reported that only a high amount of MGO inhibited *P. aeruginosa*. Honey effectively de-flagellates the bacteria and reduces its swarming and swimming capability at high concentrations of honey. However, the authors were unable to conclude whether this was due to the high level of MGO present or the complexity of honey that causes the de-flagellation. The authors further noted that, in the presence of 2 mM of MGO, *B. subtilis* and *E. coli* would lose their fimbriae and flagella. This can result in the bacteria losing their membrane integrity, resulting in shrinking that affects the ability of bacteria to swim and adhere to surfaces [43].

In this study, most of the honey samples had no inhibitory effects on *L. plantarum* at lower concentrations. Interestingly, mānuka and artificial honey at high concentrations did inhibit *L. plantarum* growth, suggesting that high concentrations of sugars may inhibit its growth. *L. plantarum* is a probiotic bacterium that inhibits the growth of undesirable bacteria in the gut by producing antibacterial substrates while competing with them for the available nutrients and bonding sites [44]. This helps stimulate the immune system by triggering antibodies and cytokines. By understanding how honey influences *L. plantarum*, we can better determine the impact of honey on the viability and therapeutic potential of probiotics. Some papers indicated that most honey would have a prebiotic effect on the gut by favouring the growth of *Lactobacilli* sp. and *Bifidobacteria* sp. [44,45,46].

### 3.2. Bacterial Growth Curves

Growth curves provide an indication of the bacterial growth dynamics. Some bacteria would have an extended lag phase of growth, which would delay their entry to the log phase of growth (exponential phase). The extension of the lag phase can impact the subsequent growth phase, as there is significant bacterial cell damage during the lag phase, preventing progression to the log phase. Understanding the bacterial growth dynamics in honey is crucial for assessing its antimicrobial properties as well as ensuring the safety and quality of honey products. Most honey samples in this study did not have an extended lag phase, except for a few cases.

Mānuka honey had a dose-dependent extended lag phase for most of the bacteria (Supplementary Figure S2). At a 25% concentration, all the bacteria had almost no growth phase. At 12.5% concentration, except for *L. plantarum* and *P. aeruginosa*, there was a significant extension of the bacteria lag phase. *S. epidermidis* and *S. aureus* experienced a lag phase of 15 and 27 h, respectively, eventually entered the log phase at 12.5% concentration, and had an extended lag phase up to 48 h at 25% concentration (Figure S2). The rest of the bacteria did not show any growth over the 48-h period, indicating growth inhibition rather than an extended lag phase. There was limited lag phase extension with the other honey samples, with the exception of some dose-dependent extension at higher concentrations. This suggests that the lag phase extension observed with mānuka honey was more pronounced compared to other honey samples. Lu, Carter [37] also found that mānuka was the most effective in extending the lag phase of all bacteria, at all concentrations. However, they found that clover and kānuka honeys did not have this effect at lower concentrations and only had some effects at higher concentrations. They attributed this extension of the lag phase to the presence of MGO and considered it a unique phenomenon of mānuka honey and other *Leptospermum* species.

There was limited lag phase extension with the other honey samples in the current study, except for some dose-dependent extension at higher concentrations. Increasing concentrations of honey considerably extended the lag phase [37]. Most bacteria did not reach a log phase at the 25% concentration of honey, except for *L. plantarum* and *P. aeruginosa*, which had an extended lag phase. At 12.5% concentration, *B. subtilis* and, *E. faecalis* had between 2 and 4 h of lag phase extension with all honey samples (Supplementary Figures S1–S8). Lu, Carter [37] also found that at high concentrations of honey, *B. subtilis* had an extended lag phase of about 16–24 h with mānuka honey and 1–4 h with kānuka and clover honey, corroborating the findings of the current study.

### 3.3. Arabinogalactan Proteins in Honey Using the Radial Gel Diffusion Technique

The presence of AGPs in the eight different samples of honey were determined (Figure S9a,b). The results showed variations in the AGP content among the different honeys tested. Pōhutukawa honey had the significantly highest amount of AGPs, followed by kāmahi, rewarewa and kānuka. Gannabathula, Krissansen [14] examined the AGP content of three types of honey (kānuka, mānuka and clover). They found that kānuka honey had significantly higher levels of AGPs compared to the other two samples, with clover honey having the least. In a similar manner, this experiment corroborates Consistent with these findings, our experiment also showed significantly higher AGP levels in kānuka honey, while clover had the lowest. Mānuka honey had significantly lower AGP levels compared to pohutukawa and kāmahi honeys. However, AGP levels were significantly higher in mānuka honey compared to honeydew and thyme honeys, as shown in Table 2. It is noteworthy that the honey samples used in our study contained significantly higher amount of AGPs compared to Gannabathula, Krissansen [14], suggesting some degree of regional effect on AGP content in different honeys, which warrants further research.

### 3.4. Antioxidant Activities, TPC and Total Flavonoid Content (TFC)

Table 3 summarises the results of the antioxidant activities, the TPC, and the TFC in different New Zealand honey samples. The complex nature of oxidative reactions can lead to inaccuracies in predicting the antioxidant capacity of honey using a single antioxidant activity assay [47]. Hence, this study employed three methodologies to improve the reliability of the experiments. Among the honey samples tested, thyme honey exhibited the highest antioxidant capacities, followed by beech honeydew, mānuka and rewarewa honey, as measured by CUPRAC, FRAP, and DPPH methods. The next highest antioxidant activities were observed in rewarewa honey, which was not significantly different from mānuka honey in terms of CUPRAC and DPPH activities. Following that were kānuka and pōhutukawa honey, which were also not significantly different from each other in terms of CUPRAC antioxidant activity. Both thyme and beech honeydew honey had higher antioxidant activity than mānuka honey. This finding is in line with a separate study by Fernández-Estellé, Hernández-González [48], which reported similar high FRAP antioxidant capacity in Spanish thyme honey. Furthermore, the FRAP assay showed that mānuka honey had a higher antioxidant activity than [16,49,50] reported for mānuka honey. The DPPH antioxidant activity of manuka honey observed in this study was consistent with previous research conducted by [17,49,51]. Moreover, a study on New Zealand honeydew honey reported comparable FRAP and CUPRAC antioxidant activities to the beech honeydew honey examined in the current study [18].

Clover and kāmahi honey had the lowest significant antioxidant activities, established using the FRAP and DPPH methods. In addition, kāmahi had the lowest significant CUPRAC activity. This difference may be due to the electronic structure and different reduction potentials of copper and ferric ions in CUPRAC and FRAP reagents. CUPRAC reagent is fast enough to oxidise thiol-type antioxidants, while other electron transfer assays based on Fe(III), such as the FRAP method, do not accurately measure certain thiol antioxidants like glutathione [52,53].

Like the antioxidant activities, thyme honey had the highest TPC, followed by beech honeydew, mānuka, rewarewa, kānuka, and pōhutukawa honey. However, kāmahi and clover had the lowest TPC values. The polyphenol content in the Folin–Ciocalteau assay is not solely attributed to the quantity of phenols present in the honey and can also involve other electron-donating antioxidants such as ascorbic acid and vitamin E. The high TPC detected in thyme honey can be attributed to its high vitamin C content (Table 4). The TPC of mānuka honey [49,51,54] was similar to the findings of this study. Additionally, Stephens, Schlothauer [55] reported similar TPC in kānuka honey in their study.

TFC was significantly the highest in beech honeydew honey, followed by mānuka, kānuka, pōhutukawa, and rewarewa. The TFC of mānuka honey in this study was similar to [56]. Thyme honey had the lowest significant TFC compared to all types of honey, except for kāmahi and clover honey. The TFC analysis using the aluminium chloride method was not influenced by the presence of ascorbic acid in honey [54,57]. Similarly, the TPC and TFC results reported in Morocco thyme honey [58] were similar to New Zealand thyme honey in this study.

### 3.5. Vitamin C Content

Under conditions of high temperature, high pH, exposure to light, and in the presence of oxygen, ascorbic acid (AA) can oxidise to form dehydroascorbic acid (DHA). This oxidation reaction is reversible, and DHA can be converted back to AA with reducing agents like dithiothreitol (DTT) [59]. The vitamin C content of the New Zealand honeys studied is shown in Table 4. Only thyme and beech honeydew New Zealand honeys contained vitamin C. The vitamin C content of Spanish thyme honey and honeydew honey was similar to our findings [33]. Previous research on Romanian honeydew honey reported a range of vitamin C content, and our results for beech honeydew fall within this range [60]. In thyme honey, the total vitamin C content was 425 ± 6.10 mg/kg, with an AA content of 383 ± 3.41 and DHA content of 42. However, no oxidised product of AA (DHA) was detected in beech honeydew due to its low concentration. Vitamin C can serve as a chemical marker to distinguish New Zealand thyme honey from other varieties, as it is found in high quantities only in thyme honey. Vitamin C is a potent antioxidant and can be detected using various assays such as FRAP, CUPRAC, DPPH, and Folin–Ciocalteau TPC [52,61,62]. Therefore, the higher vitamin C content in thyme honey likely contributes to its superior antioxidant activity and TPC compared to other New Zealand honeys in the current study.

### 3.6. PLS-DA Analysis of Antioxidants, Total Phenolic, Total Flavonoid, Ascorbic and Vitamin C in Honey Samples

**Figure 2:**
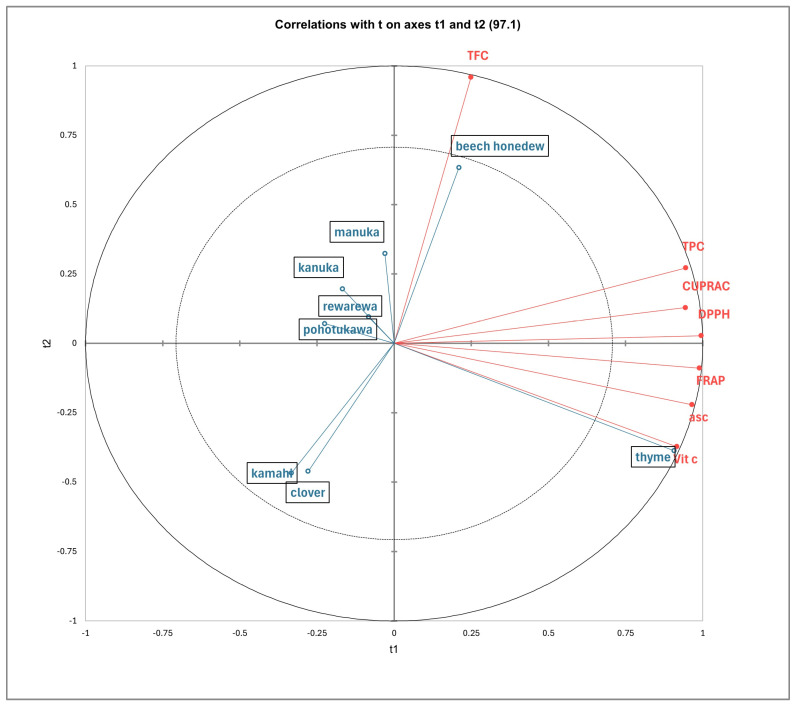
Partial least squares discriminant analysis of New Zealand honey based on antioxidants (CUPRAC, DPPH and FRAP), total phenolic content (TPC), total flavonoid content (TFC), ascorbic acid content (asc), and vitamin C content.

The PLS-DA model indicated the relationship between the honey samples and the polyphenols, as shown in Figure 2. The more acute the angle between the sample vectors and the polyphenol vectors, the stronger the positive correlation is between them [63]. Conversely, the more obtuse the angle between the two vectors is, the weaker the correlation. Additionally, the length of the vector arrows indicated the level of positive correlation, with the longer arrows representing stronger correlations. The first two latent components—t1 and t2—collectively explained 97.1% of the variance, meaning they capture almost all the important information (Figure 2).

Thyme honey was closely correlated with the TPC, CUPRAC, DPPH, and FRAP, as well as ascorbic acid and vitamin C content, which had high positive loadings along t1. Thyme honey had the highest significant antioxidant capacities (Table 3) and vitamin C (Table 4), followed by beech honeydew, compared to the other honey samples. Beech honeydew was highly correlated to TFC, with a high positive loading along t2. This finding is supported by the results shown in Table 3, which show that beech honeydew honey has significantly higher TFC compared to all other honey samples.

### 3.7. Polyphenol Analysis of Honeys Using LC-MS

Polyphenol content in the different New Zealand honey samples is summarised in Table 5. There were twelve polyphenols present in varying concentrations in all honey samples: quinic acid, hydroxybenzoic acid, caffeic acid, p-coumaric acid, rutin, benzoic acid, luteolin, quercetin, kaempferol, pinobanksin, chrysin, and pinocembrin. Gallic acid was only present in mānuka, pōhutukawa and kāmahi honeys.

Pinocembrin was the main polyphenol present at the highest concentration in all samples, with the highest significant level found in the clover honey sample. Pinobanksin was the next highest polyphenol present in all samples, with the highest significant level found in the clover honey, kanuka, rewarewa, and pohutukawa honey samples. Similar findings were reported by Deadman [19], Weston, Brocklebank and Lu [23] and Yao, Datta [21], who identified pinocembrin and pinobanksin as major polyphenols present in both New Zealand mānuka and non-mānuka varieties. Weston, Brocklebank and Lu [23] suggested that these polyphenols are commonly derived from propolis and are frequently found in honeys from temperate climates in the northern hemisphere [19,20,21,23]

Caffeic acid, p-coumaric, quercetin and kaempferol were present at high concentrations in clover honey. Additionally, pinobanksin, and pinocembrin were significantly high in clover honey compared to other honeys.

Mānuka honey had the highest significant concentration of luteolin—almost three times more than the other honey samples. Deadman [19] reported that mānuka honey could be distinguished from other honey varieties based on its high amount of luteolin. Yao, Datta [21] found that New Zealand mānuka and Australian Jelly bush honey (also from the *Leptospermum* family, *Leptospermum polygalifolium*) had a common flavonoid profile that included quercetin, quercetin 3-methyl ether, luteolin, and an unknown flavonoid. This study also showed significant amounts of both chrysin and gallic acid in mānuka honey. In addition, gallic acid was only present in the mānuka, honeydew, pōhutukawa and kāmahi honey samples. High amounts of chrysin, gallic acid, and abscisic acid have been reported to differentiate New Zealand mānuka honey from Australian Jelly bush honey, which contains high amounts of myricetin [21]

Honeydew honey had the highest significant amount of hydroxybenzoic acid and quinic acid compared to other honey samples. In addition, it contained a significantly higher level of benzoic acid compared to most honey samples, except for thyme honey, which exhibited the highest concentration of benzoic acid among all samples tested. Weston, Brocklebank and Lu [23] also reported high concentrations of hydroxybenzoic acid and benzoic acid in honeydew honey compared to clover and mānuka honey. Pōhutukawa honey had the highest significant amount of gallic acid. It also had significant amounts of caffeic acid and quercetin, like kānuka honey. Kāmahi honey had the highest significant concentration of rutin, along with elevated levels of caffeic acid and benzoic acid. Thyme honey had the highest significant amount of benzoic acid. Pauliuc, Dranca and Oroian [60] also found that Romanian thyme had high concentrations of hydroxybenzoic acid, p-coumaric acid and caffeic acid but did not contain quercetin, luteolin and kaempferol, which were present in the New Zealand thyme honey sample. In Table 5, the F-values associated with each polyphenol indicate the extent to which the mean concentrations differ across the various honey samples. A higher F-value indicates a greater degree of difference between the group means. Conversely, the *p*-values for each polyphenol indicate the significance of differences in their concentrations among the honey samples. A lower *p*-value indicates that the observed differences are more likely to be statistically significant.

### 3.8. PLS-DA Analysis of Phenolic Content in Honey Samples

As in ref. [63], along t1, clover honey had high positive scores and was found to be strongly correlated to kaempferol, pinobanksin, and pinocembrin. Interestingly, Chan, Deadman [20] found that mānuka honey had high amounts of pinobanksin, pinocembrin, chrysin and luteolin, which accounted for 61% of the total phenolic content. Although mānuka honey also contained significant amounts of all these four polyphenols in the current study, clover honey had the highest amounts of pinocembrin, pinobanksin and chrysin (Table 5).

Thyme honey had a high negative score along t1 and was strongly correlated to benzoic acid. It can be further observed that mānuka and pōhutukawa honey had high negative scores along t2 and were strongly correlated to luteolin, quercetin and gallic acid. Stephens, Schlothauer [55] also found that mānuka and kānuka honey samples contained higher luteolin, gallic acid and quercetin content [55]. Similarly, Yao, Datta [21] found that mānuka honey had a high amount of luteolin, chrysin, gallic acid and quercetin.

**Figure 3:**
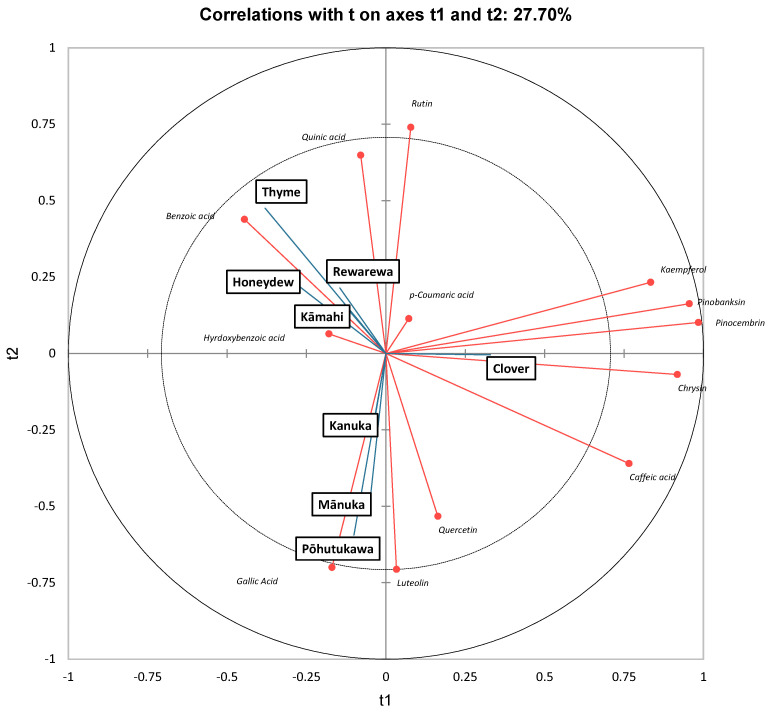
Partial least squares discriminant analysis of New Zealand honeys, based on polyphenolic content.

**Figure 4:**
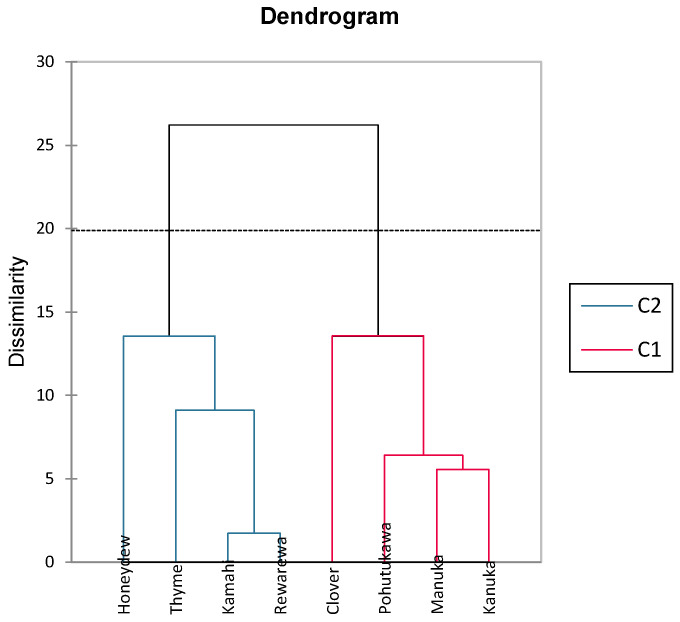
Dendrogram cluster analysis showing phenolic dissimilarity in different New Zealand honeys.

The dendrogram shown in Figure 3 is an output from an agglomerative hierarchical cluster analysis that illustrates how closely related the honey samples are taxonomically in terms of their polyphenolic content. This is determined by the length of connecting lines, with shorter lines indicating a closer relationship [64]. As shown in Figure 4, it can be seen that kāmahi and rewarewa honey are closely related, as are kānuka and mānuka honey. Stephens, Schlothauer [55] indicated that mānuka and kānuka were similar in terms of polyphenol content, as most mānuka bushes (*L. scoparium*) are interspersed with kānuka bushes (*K. ericoides*), making it hard to achieve monoflorality. In addition, it is also hard to differentiate the pollen between both these plants as they look identical, making it tough to differentiate these two honey samples [55]. Thyme and honeydew honey were closely related to kāmahi and rewarewa, as seen in Figure 4. Pohutukawa and clover honey were also closely related to mānuka and kānuka.

## 4. Conclusions

Given the growing interest in the health benefits of honey, conducting further research on the bioactive properties of New Zealand native honeys, beyond mānuka honey, is essential to fully understand their potential impact on human health. This study aimed to investigate the antibacterial properties, arabinogalactan proteins (AGPs), antioxidant activities, and polyphenolic content of eight different types of New Zealand honey. The results revealed that the honey types varied in their antibacterial activity, with mānuka, pōhutukawa, and kāmahi honey showing significant inhibitory effects against bacteria. Moreover, all honey samples exhibited inhibitory effects on bacterial growth at a concentration of 25%. AGPs were found in all eight honey samples, with higher amounts detected in pōhutukawa, kāmahi, rewarewa, kānuka, and mānuka honey. Thyme honey demonstrated the highest antioxidant capacities, followed by beech honeydew and mānuka honey. Beech honeydew had the highest total flavonoid content, while kāmahi and clover honey had the lowest total phenolic content. It is interesting to note that only thyme and beech honeydew New Zealand honeys contained vitamin C. Different honey types exhibited varying concentrations of polyphenols, with mānuka, kānuka, and pōhutukawa honeys containing high levels of quercetin, luteolin, and gallic acid, respectively, and clover honey showing notable levels of chrysin, pinocembrin, caffeic acid, and pinobanksin. This study provides valuable information regarding the antibacterial properties and bioactivities of native New Zealand honeys, which is particularly noteworthy as most research in the field has predominantly focused on mānuka honey. The findings contribute to our understanding of the potential health benefits associated with these honeys and suggest their potential use as natural alternatives to enhance human health.

## Figures and Tables

**Table 1:** Broth dilution results showing the susceptibility of microorganisms to various honey samples. Results were analysed using Analysis of Covariance (ANCOVA). The values represent Least Squares Means (LSMeans), which are the adjusted means of the optical density (OD) for each honey concentration (25, 12.5, 6.25 and 3.13% *w*/*v*), after accounting for the covariate (time). Fisher’s Least Significant Difference (LSD) test was used as a post-hoc analysis following the ANCOVA at the 5% level. Superscripts with different letters indicate that the LSMeans within each column for each honey are significantly different.

	Bacteria	*E.* *coli*	*B. subtilis*	*S. typhimurium*	*E. faecalis*	*P. aeruginosa*	*S. epidermidis*	*S. aureus*	*L. plantarum*
**Clover Honey**	**25%**	0.143 ^c^	0.081 ^d^	0.088 ^d^	0.192 ^c^	0.755 ^c^	0.946 ^b^	0.297 ^c^	1.782 ^b^
	**12.50%**	0.213 ^c^	0.249 ^c^	0.141 ^bc^	0.339 ^ab^	1.702 ^b^	0.999 ^b^	0.842 ^b^	1.899 ^b^
	**6.25%**	0.294 ^b^	0.400 ^a^	0.165 ^b^	0.317 ^b^	2.341 ^a^	1.596 ^a^	1.024 ^a^	2.037 ^ab^
	**3.13%**	0.329 ^b^	0.330 ^b^	0.134 ^c^	0.295 ^b^	2.481 ^a^	1.657 ^a^	1.016 ^a^	1.977 ^ab^
	**0%**	0.638 ^a^	0.288 ^bc^	0.221 ^a^	0.362 ^a^	1.673 ^b^	1.557 ^a^	0.832 ^b^	2.259 ^a^
**Mānuka Honey**	**25%**	0.062 ^c^	0.046 ^c^	0.070 ^c^	0.166 ^c^	0.300 ^c^	0.322 ^c^	0.343 ^b^	0.770 ^b^
	**12.50%**	0.058 ^c^	0.062 ^c^	0.063 ^c^	0.137 ^c^	1.734 ^b^	0.757 ^b^	0.491 ^b^	2.003 ^a^
	**6.25%**	0.264 ^b^	0.177 ^b^	0.080 ^bc^	0.244 ^b^	2.351 ^a^	1.387 ^a^	0.902 ^a^	1.957 ^a^
	**3.13%**	0.305 ^b^	0.327 ^a^	0.098 ^b^	0.266 ^b^	2.244 ^a^	1.598 ^a^	0.929 ^a^	2.005 ^a^
	**0%**	0.638 ^a^	0.288 ^a^	0.221 ^a^	0.362 ^a^	1.673 ^b^	1.557 ^a^	0.832 ^a^	2.259 ^a^
**Honeydew Honey**	**25%**	0.149 ^d^	0.324 ^d^	0.081 ^d^	0.206 ^c^	1.244 ^c^	0.398 ^c^	0.351 ^b^	1.740 ^b^
	**12.50%**	0.289 ^c^	1.204 ^bc^	0.132 ^c^	0.281 ^b^	2.088 ^a^	1.249 ^b^	0.794 ^a^	1.843 ^b^
	**6.25%**	0.675 ^a^	1.464 ^a^	0.139 ^bc^	0.301 ^b^	2.211 ^a^	1.669 ^a^	0.891 ^a^	1.972 ^b^
	**3.13%**	0.410 ^b^	1.418 ^ab^	0.162 ^b^	0.274 ^b^	1.996 ^ab^	1.634 ^a^	0.821 ^a^	1.988 ^ab^
	**0%**	0.723 ^a^	1.185 ^c^	0.221 ^a^	0.362 ^a^	1.673 ^b^	1.557 ^a^	0.891 ^a^	2.259 ^a^
**Pōhutukawa Honey**	**25%**	0.165 ^c^	0.382 ^b^	0.081 ^b^	0.130 ^c^	0.499 ^b^	0.534 ^d^	0.466 ^c^	1.688 ^b^
	**12.50%**	0.268 ^b^	1.284 ^a^	0.136 ^b^	0.205 ^b^	1.598 ^a^	1.359 ^bc^	0.947 ^b^	1.847 ^ab^
	**6.25%**	0.336 ^b^	1.299 ^a^	0.163 ^b^	0.196 ^b^	1.424 ^a^	1.584 ^ab^	1.047 ^b^	1.946 ^ab^
	**3.13%**	0.344 ^b^	1.316 ^a^	0.186 ^b^	0.204 ^b^	1.399 ^a^	1.613 ^a^	1.267 ^a^	1.882 ^ab^
	**0%**	0.723 ^a^	1.185 ^a^	1.396 ^a^	0.233 ^a^	1.422 ^a^	1.112 ^c^	1.095 ^b^	2.111 ^a^
**Kānuka Honey**	**25%**	0.175 ^d^	0.096 ^c^	0.107 ^b^	0.143 ^d^	0.707 ^c^	0.688 ^c^	0.336 ^c^	1.553 ^b^
	**12.50%**	0.263 ^c^	0.074 ^c^	0.175 ^b^	0.209 ^c^	1.693 ^ab^	1.244 ^b^	0.795 ^b^	1.947 ^a^
	**6.25%**	0.340 ^b^	0.361 ^a^	0.190 ^b^	0.255 ^a^	1.981 ^a^	1.073 ^b^	1.061 ^a^	1.940 ^a^
	**3.13%**	0.370 ^b^	0.398 ^a^	0.173 ^b^	0.229 ^bc^	1.445 ^b^	1.665 ^a^	0.908 ^b^	1.991 ^a^
	**0%**	0.638 ^a^	0.288 ^b^	1.396 ^a^	0.233 ^ab^	1.422 ^b^	1.112 ^b^	1.095 ^a^	2.111 ^a^
**Rewarewa Honey**	**25%**	0.128 ^d^	0.088 ^c^	0.160 ^b^	0.166 ^c^	0.860 ^d^	0.903 ^c^	0.483 ^d^	2.050 ^a^
	**12.50%**	0.244 ^c^	0.320 ^ab^	0.155 ^b^	0.219 ^b^	1.853 ^a^	1.467 ^ab^	0.803 ^c^	2.098 ^a^
	**6.25%**	0.305 ^bc^	0.326 ^ab^	0.180 ^b^	0.216 ^b^	1.706 ^ab^	1.677 ^a^	0.846 ^bc^	1.985 ^a^
	**3.13%**	0.343 ^b^	0.352 ^a^	0.188 ^b^	0.258 ^a^	1.496 ^bc^	1.448 ^b^	0.969 ^ab^	1.975 ^a^
	**0%**	0.638 ^a^	0.288 ^b^	1.396 ^a^	0.233 ^b^	1.422 ^c^	1.112 ^c^	1.095 ^a^	2.111 ^a^
**Kāmahi Honey**	**25%**	0.167 ^d^	0.519 ^c^	0.129 ^b^	0.205 ^c^	0.739 ^c^	0.979 ^c^	0.612 ^d^	1.928 ^a^
	**12.50%**	0.284 ^c^	1.433 ^a^	0.167 ^b^	0.233 ^b^	1.874 ^a^	1.429 ^b^	0.794 ^c^	1.916 ^a^
	**6.25%**	0.337 ^c^	1.427 ^a^	0.173 ^b^	0.226 ^bc^	1.684 ^ab^	1.597 ^ab^	0.900 ^bc^	1.959 ^a^
	**3.13%**	0.428 ^b^	1.274 ^ab^	0.195 ^b^	0.258 ^a^	1.474 ^b^	1.727 ^a^	0.964 ^ab^	1.975 ^a^
	**0%**	0.723 ^a^	1.185 ^b^	1.396 ^a^	0.233 ^b^	1.422 ^b^	1.112 ^c^	1.095 ^a^	2.111 ^a^
**Thyme Honey**	**25%**	0.339 ^c^	0.291 ^c^	0.310 ^c^	0.186 ^c^	0.901 ^c^	0.656 ^c^	0.278 ^c^	1.753 ^b^
	**12.50%**	1.078 ^b^	1.028 ^a^	0.647 ^b^	0.246 ^b^	2.092 ^a^	1.526 ^ab^	0.688 ^b^	1.955 ^ab^
	**6.25%**	1.282 ^a^	1.108 ^a^	0.872 ^a^	0.232 ^b^	1.797 ^a^	1.771 ^a^	0.804 ^b^	1.925 ^ab^
	**3.13%**	1.317 ^a^	1.049 ^a^	0.878 ^a^	0.323 ^a^	1.490 ^b^	1.653 ^a^	0.948 ^a^	1.962 ^ab^
	**0%**	0.999 ^b^	0.736 ^b^	0.604 ^b^	0.297 ^a^	1.548 ^b^	1.335 ^b^	0.964 ^a^	2.185 ^a^
**Artificial Honey**	**25%**	0.657 ^b^	0.495 ^c^	0.899 ^a^	0.202 ^c^	1.193 ^b^	0.960 ^b^	0.916 ^c^	1.737 ^c^
	**12.50%**	0.317 ^d^	0.613 ^b^	0.740 ^b^	0.206 ^c^	1.787 ^a^	1.245 ^a^	1.064 ^bc^	2.017 ^bc^
	**6.25%**	0.493 ^c^	0.637 ^ab^	0.816 ^ab^	0.273 ^b^	1.800 ^a^	1.286 ^a^	1.142 ^ab^	2.216 ^ab^
	**3.13%**	0.565 ^bc^	0.725 ^a^	0.883 ^a^	0.341 ^a^	1.598 ^a^	1.305 ^a^	1.297 ^a^	2.381 ^a^
	**0%**	0.999 ^a^	0.736 ^a^	0.604 ^c^	0.297 ^b^	1.548 ^a^	1.335 ^a^	0.964 ^bc^	2.185 ^ab^

**Table 2:** Radial gel diffusion results showing the mean amount of arabinogalactan proteins (AGPs) present in different honey samples. Superscripts that have different letters indicate that the means are significantly different at the 5% level based on Fisher’s LSD.

Samples	AGPs (µg/gram) (Honey/Buffer 1:1)
**Clover**	1198.754 ^de^
**Mānuka**	1479.273 ^cd^
**Honeydew**	1071.936 ^e^
**Pōhutukawa**	3222.133 ^a^
**Kānuka**	1588.035 ^bcd^
**Rewarewa**	1824.695 ^bc^
**Kāmahi**	1903.015 ^b^
**Thyme**	1024.599 ^e^
**Pr > F**	*<0.0001*

The unit of measurement is expressed as micrograms of gum Arabic per gram of honey (µg/g).

**Table 3:** Antioxidant activities, as well as total phenolic and flavonoid contents, in honey (means ± standard error).

Honey	CUPRAC Antioxidant Capacity (mg TEAC/kg Honey)	FRAP Antioxidant Capacity (mg TEAC/kg Honey)	DPPH· Antioxidant Capacity (mg TEAC/kg Honey)	TPC Content (mg GAE/kg Honey)	TFC Content (mg RUE/kg Honey)
Clover	871 ± 3.29 ^d^	330 ± 8.81 ^g^	117 ± 1.04 ^f^	380 ± 5.57 ^h^	97 ± 1.33 ^g^
Mānuka	908 ± 1.90 ^c^	648 ± 3.52 ^c^	252 ± 1.65 ^c^	726 ± 4.76 ^c^	192 ± 3.38 ^b^
Beech honeydew	1264 ± 5.17 ^b^	753 ± 1.10 ^b^	377 ± 4.61 ^b^	809 ± 6.41 ^b^	235 ± 3.38 ^a^
Pohotukawa	754 ± 8.08 ^e^	427 ± 6.10 ^f^	143 ± 0.52 ^e^	463 ± 7.49 ^f^	172 ± 2.88 ^d^
Kānuka	745 ± 4.48 ^e^	516 ± 1.76 ^e^	175 ± 1.09 ^d^	558 ± 7.13 ^e^	186 ± 2.39 ^c^
Rewa-rewa	905 ± 2.87 ^c^	543 ± 9.15 ^d^	244 ± 2.22 ^c^	652 ± 1.06 ^d^	163 ± 1.95 ^e^
Kāmahi	540 ± 4.97 ^f^	345 ± 8.07 ^g^	133 ± 1.15 ^f^	398 ± 7.49 ^g^	101 ± 2.24 ^g^
Thyme	1564 ± 5.17 ^a^	1731 ± 14.44 ^a^	664 ± 1.81 ^a^	1058 ± 8.39 ^a^	147 ± 2.88 ^f^
F value	13,517.244	7914.448	2223.254	2762.378	885.258
*p*-value	<0.0001	<0.0001	<0.0001	<0.0001	<0.0001

Data are expressed as mean ± standard deviation (n = 3). Values in each column with different superscript letters differ significantly (fisher test, *p* < 0.05).

**Table 4:** Results for vitamin C analysis in honey using HPLC (means ± standard error).

Honey	Total Vitamin C (mg/kg Honey)	Ascorbic Acid (mg/kg Honey)	DHA (mg/kg Honey)
Clover	Nd	Nd	Nd
Mānuka	Nd	Nd	Nd
Beech honeydew	11 ± 0.55	11 ± 0.55	Nd
Pōhutukawa	Nd	Nd	Nd
Kānuka	Nd	Nd	Nd
Rewarewa	Nd	Nd	Nd
Kāmahi	Nd	Nd	Nd
Thyme	425 ± 6.10	383 ± 3.41	42
F value	14,250.871	37,860.581	-
*p*-value	<0.0001	<0.0001	-

Nd—not detected.

**Table 5:** Polyphenols in New Zealand honey samples. Mean concentrations are expressed in μg/mL. Superscripts that have different letters indicate that the means are significantly different at the 5% level based on Fisher’s LSD.

	Quinic Acid	Gallic Acid	Hydroxybenzoic Acid	Caffeic Acid	p-Coumaric Acid	Rutin	Benzoic Acid	Luteolin	Quercetin	Kaempferol	Pinobanksin	Chrysin	Pinocembrin
**Clover**	0.933 ^bcd^	0.000 ^d^	2.865 ^bc^	0.730 ^a^	0.429 ^a^	0.023 ^d^	0.328 ^c^	0.536 ^cd^	0.107 ^a^	0.357 ^a^	2.627 ^a^	0.871 ^a^	8.200 ^a^
**Mānuka**	0.725 ^bcd^	0.161 ^b^	3.386 ^b^	0.553 ^abc^	0.061 ^f^	0.023 ^d^	0.343 ^c^	3.671 ^a^	0.098 ^abc^	0.150 ^bc^	1.854 ^b^	0.868 ^a^	5.531 ^b^
**Honeydew**	1.729 ^a^	0.088 ^c^	10.003 ^a^	0.520 ^bc^	0.144 ^de^	0.043 ^c^	0.653 ^b^	0.616 ^cd^	0.096 ^abc^	0.135 ^c^	1.766 ^b^	0.684 ^ab^	5.032 ^b^
**Pōhutukawa**	0.538 ^d^	0.462 ^a^	2.243 ^cd^	0.622 ^abc^	0.241 ^c^	0.024 ^d^	0.147 ^d^	1.557 ^b^	0.103 ^ab^	0.166 ^bc^	1.956 ^ab^	0.567 ^bc^	4.726 ^b^
**Kānuka**	0.632 ^cd^	0.000 ^d^	2.230 ^cd^	0.627 ^ab^	0.185 ^cd^	0.016 ^d^	0.248 ^cd^	0.934 ^c^	0.088 ^abc^	0.121 ^c^	2.033 ^ab^	0.657 ^ab^	5.588 ^b^
**Rewarewa**	1.214 ^ab^	0.000 ^d^	1.790 ^cd^	0.438 ^cd^	0.148 ^de^	0.054 ^bc^	0.206 ^cd^	0.192 ^d^	0.075 ^abc^	0.139 ^bc^	2.014 ^ab^	0.554 ^bc^	4.712 ^b^
**Kāmahi**	1.020 ^bcd^	0.089 ^c^	2.326 ^bcd^	0.584 ^abc^	0.102 ^ef^	0.072 ^a^	0.207 ^cd^	0.613 ^cd^	0.065 ^c^	0.129 ^c^	1.790 ^b^	0.540 ^bc^	5.268 ^b^
**Thyme**	1.130 ^bc^	0.000 ^d^	1.494 ^d^	0.318 ^d^	0.328 ^b^	0.067 ^ab^	0.827 ^a^	0.661 ^c^	0.065 ^bc^	0.207 ^b^	1.531 ^b^	0.392 ^c^	3.504 ^b^
F value	3.860	48.260	51.500	4.240	26.950	20.750	20.710	52.980	1.880	17.770	16.020	10.050	12.850
*p*-value	0.010	<0.0001	<0.0001	0.007	<0.0001	<0.0001	<0.0001	<0.0001	0.136	<0.0001	<0.0001	<0.0001	<0.0001

## Data Availability

Data is contained within the article and Supplementary Materials.

## Supplementary Materials

The following supporting information can be downloaded at: http://www.mdpi.com/article/10.3390/antiox14040375/s1. Table S1 Liquid chromatography mass spectrometry MRM transitions. Figure S1: Broth Microdilution Assay growth curves with clover honey. Figure S2: Broth Microdilution Assay growth curves with mānuka honey. Figure S3: Broth Microdilution Assay growth curves with honeydew honey. Figure S4: Broth Microdilution Assay growth curves with pōhutukawa honey. Figure S5: Broth Microdilution Assay growth curves with kānuka honey. Figure S6: Broth Microdilution Assay growth curves with kāmahi honey. Figure S7: Broth Microdilution Assay growth curves with thyme honey. Figure S8: Broth Microdilution Assay growth curves with artificial honey. Figure S9a. AGP diffusion gel assay of honeys; from left to right Arabic controls of 1.25 mg, 2.5 mg, 5 mg, Clover, Manuka, Honeydew and Pohutukawa. Figure S9b. AGP diffusion gel assay of honeys; from left to right Arabic controls of 1.25 mg, 2.5 mg, 5 mg, Kanuka, Rewarewa, Kamahi and Thyme.
