# Supplementary material for: Antibacterial Properties, Arabinogalactan Proteins, and Bioactivities of New Zealand Honey
Source: Antioxidants (Basel). 2025 Mar 21;14(4):375. doi: 10.3390/antiox14040375 (PMC12023948; doi:10.3390/antiox14040375)
Supplement: Supplementary file 1 [file antioxidants-14-00375-s001.zip › antioxidants-3326115-supplementary.pdf]

Supplementary Table S1

Liquid chromatography mass spectrometry MRM transitions

Compound name	RT (min)	Molecular Formula	R^2	Precursor Ion	Product Ion	Fragmentor	Collision Energy	Cell Accelerator Voltage	Polarity
Apigenin 7-o-glucoside	20.4671	$C_{21}H_{20}O_{10}$	y=60.3130x+697.9966	431	431	220	0	7	Negative
			R^2=0.9984	431	268	220	33	7	Negative
Benzoic acid	19.8129	$C_7H_6O_6$	y= 1.4087x – 23.7276	121	121	100	0	7	Negative
			R^2=0.9979	121	77	100	8	7	Negative
Caffeic acid	12.6685	$C_9H_8O_4$	y= 33.3529x +722.820	179	135	80	13	7	Negative
			R^2= 0.9980	179	134	80	34	7	Negative
Catechin	11.8525	$C_{15}H_{14}O_6$	y=4.1913x- 21.0171	289	245	140	6	7	Negative
			R^2=0.9990	289	203	140	12	7	Negative
Chrysin	26.1441	$C_{15}H_{11}O_4$	y= 8.4415x + 25.9013	253	143	160	24	7	Negative
			R^2= 0.9997	253	119	160	32	7	Negative
Gallic acid	2.3883	$C_7H_6O_5$	y=2.7894x -57.457	169	169	200	0	7	Negative
			R^2= 0.999	169	128	200	10	7	Negative
				169	79	200	24	7	Negative
Homovanillic acid	13.9163	$C_9H_{10}O_4$	y=0.9153x+2.2748	181	137	80	1	7	Negative
			R2=0.9986	181	122	80	9	7	Negative
Hydroxybenzoic acid	7.9288	$C_7H_6O_3$	y= 5.1567x +209.097 R^2= 0.9968	137	137	80	0	7	Negative
Kaempferol	22.8312	$C_{15}H_{10}O_6$	y= 101.8595x	285	285	140	0	7	Negative
			R^2= 0.9962	285	185	140	23	7	Negative
				285	156	140	26	7	Negative
Luteolin	21.769	$C_{15}H_{10}O_6$	y= 3.844x +26.456	285	133	180	33	7	Negative

			R^2= 0.9989						
p-coumaric acid	17.4594	$C_9H_8O_3$	y=36.9065x +251.490 R^2= 0.9997	163	119	80	12	7	Negative
				163	93	80	36	7	Negative
Pinobanksin	22.9408	$C_{15}H_{13}O_5$ C_{15} $H_{11}O_5$	y= 13.2323x + 203.1724 R^2= 0.9985	271	253	140	15	7	Negative
				271	197	140	20	7	Negative
Pinocembrin	26.6667	$C_{15}H_{12}O_4$	y= 3.708x -5.674 R^2= 0.9992	255	213	140	16	7	Negative
				255	107	140	26	7	Negative
Quercetin	21.8163	$C_{15}H_{10}O_7$	y= 35.3454x R^2= 0.9968	301	179	140	10	7	Negative
				301	151	140	16	7	Negative
Quinic acid	1.2908	$C_7H_{12}O_6$	y= 17.0651x +297.7674 R^2= 0.9978	191	191	160	0	7	Negative
Rutin	19.6387	$C_{27}H_{30}O_{16}$	y=11.207x +77.719 R^2= 0.9992	609	271	240	64	7	Negative
				609	300	240	40	7	Negative

Supplementary Figure S1.

Broth Microdilution Assay growth curves with clover honey

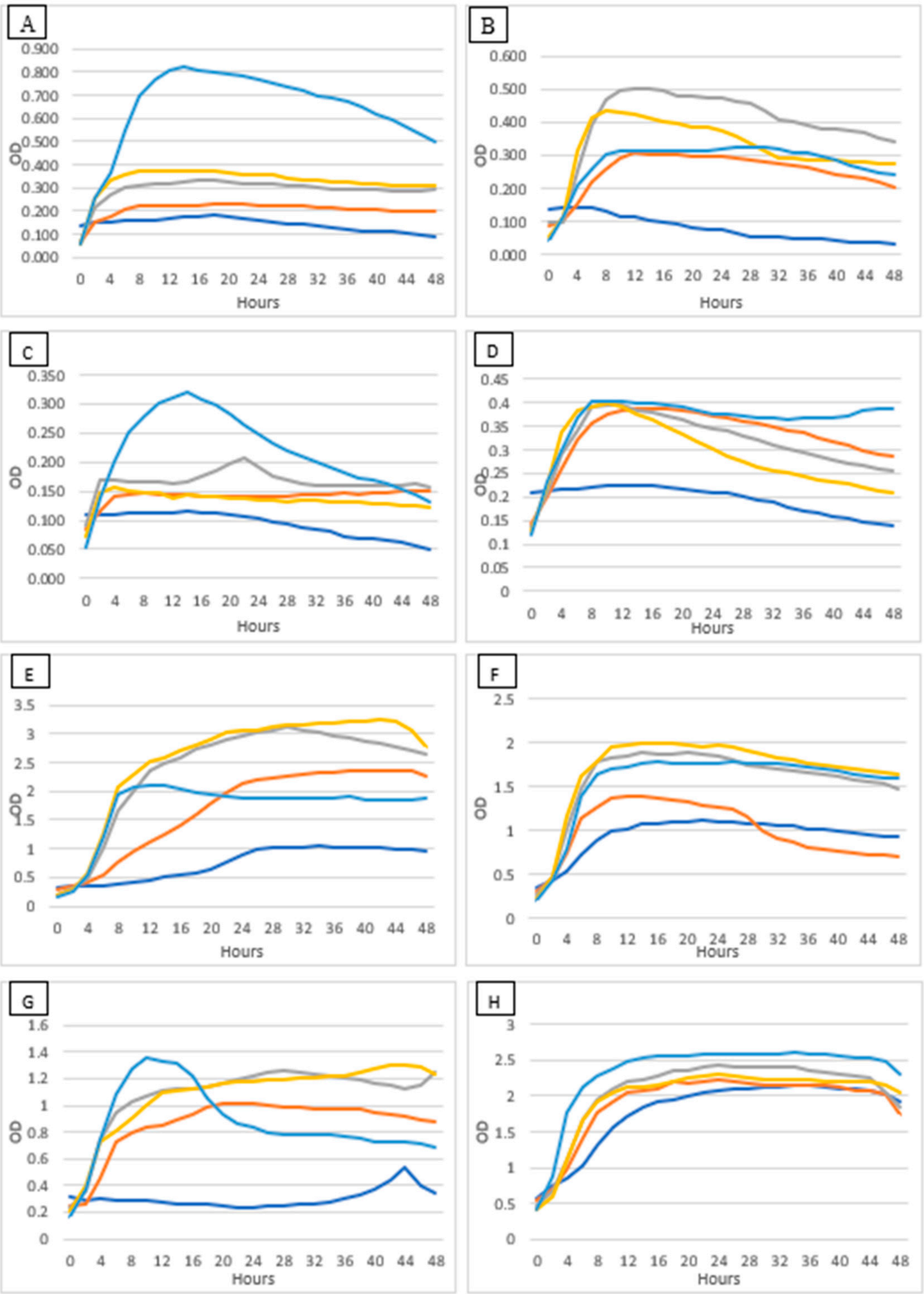

Clover honey was tested with eight bacteria at four concentrations and a control. Corresponding graphs a) *E. coli* b) *B. subtilis* c) *S. typhimurium* d) *E. faecalis* e) *P. aeruginosa* f) *S. epidermidis* g) *S. aureus* h) *L. plantarum*. Corresponding concentration levels

25.00% 12.50% 6.25% 3.13% Positive control

Supplementary Figure S2

Broth Microdilution Assay growth curves with mānuka honey

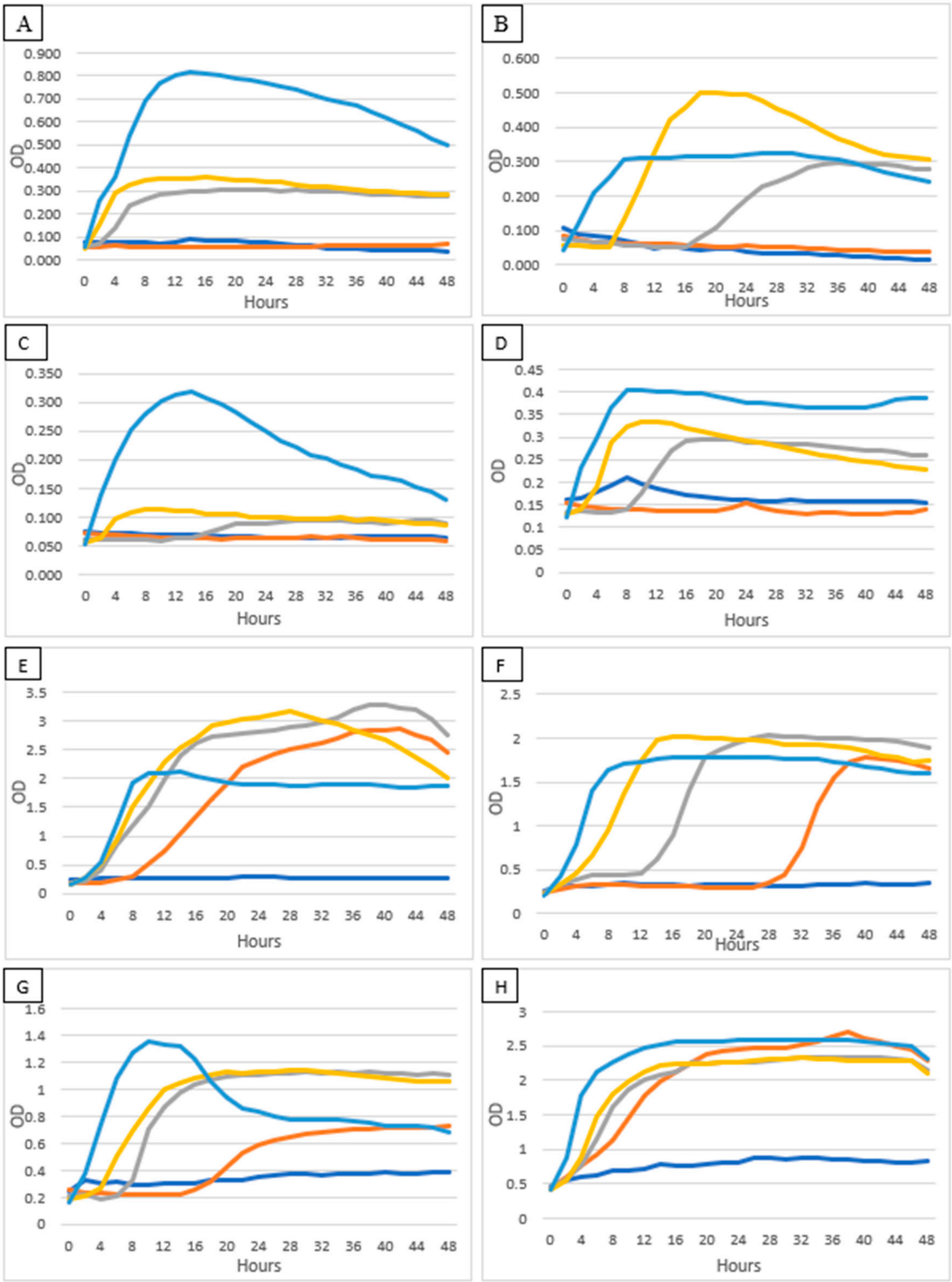

Mānuka honey was tested with eight bacteria at four concentrations and a control. Corresponding graphs a) *E. coli* b) *B. subtilis* c) *S. typhimurium* d) *E. faecalis* e) *P. aeruginosa* f) *S. epidermidis* g) *S. aureus* h) *L. plantarum*. Corresponding concentration levels 25.00% 12.50% 6.25% 3.13% Positive control

Supplementary Figure S3

Broth Microdilution Assay growth curves with honeydew honey

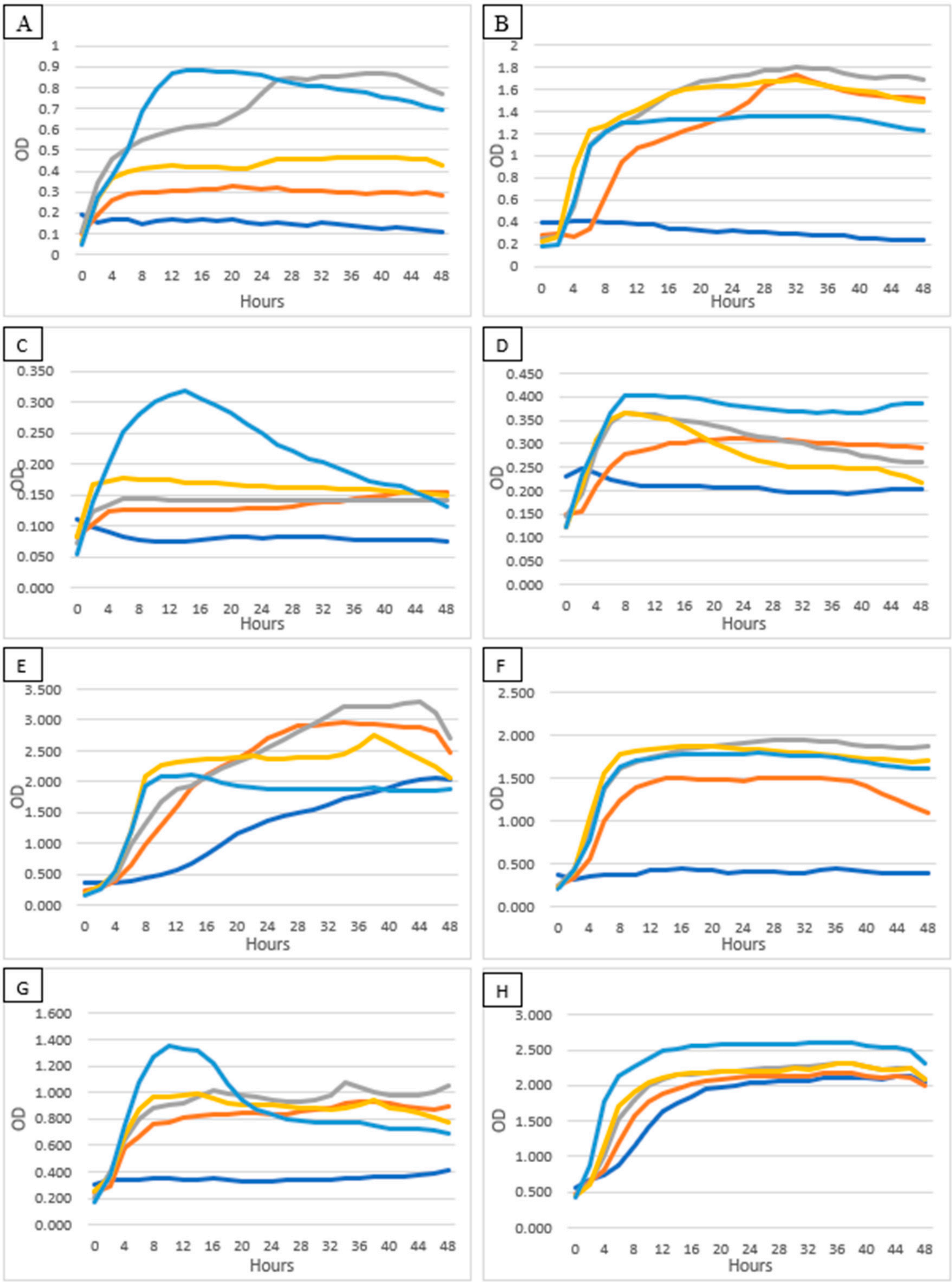

Honeydew honey was tested with eight bacteria at four concentrations and a control. Corresponding graphs a) *E. coli* b) *B. subtilis* c) *S. typhimurium* d) *E. faecalis* e) *P. aeruginosa* f) *S. epidermidis* g) *S. aureus* h) *L. plantarum*. Corresponding concentration levels 25.00% 12.50% 6.25% 3.13% Positive control

Supplementary Figure S4

Broth Microdilution Assay growth curves with pōhutukawa honey

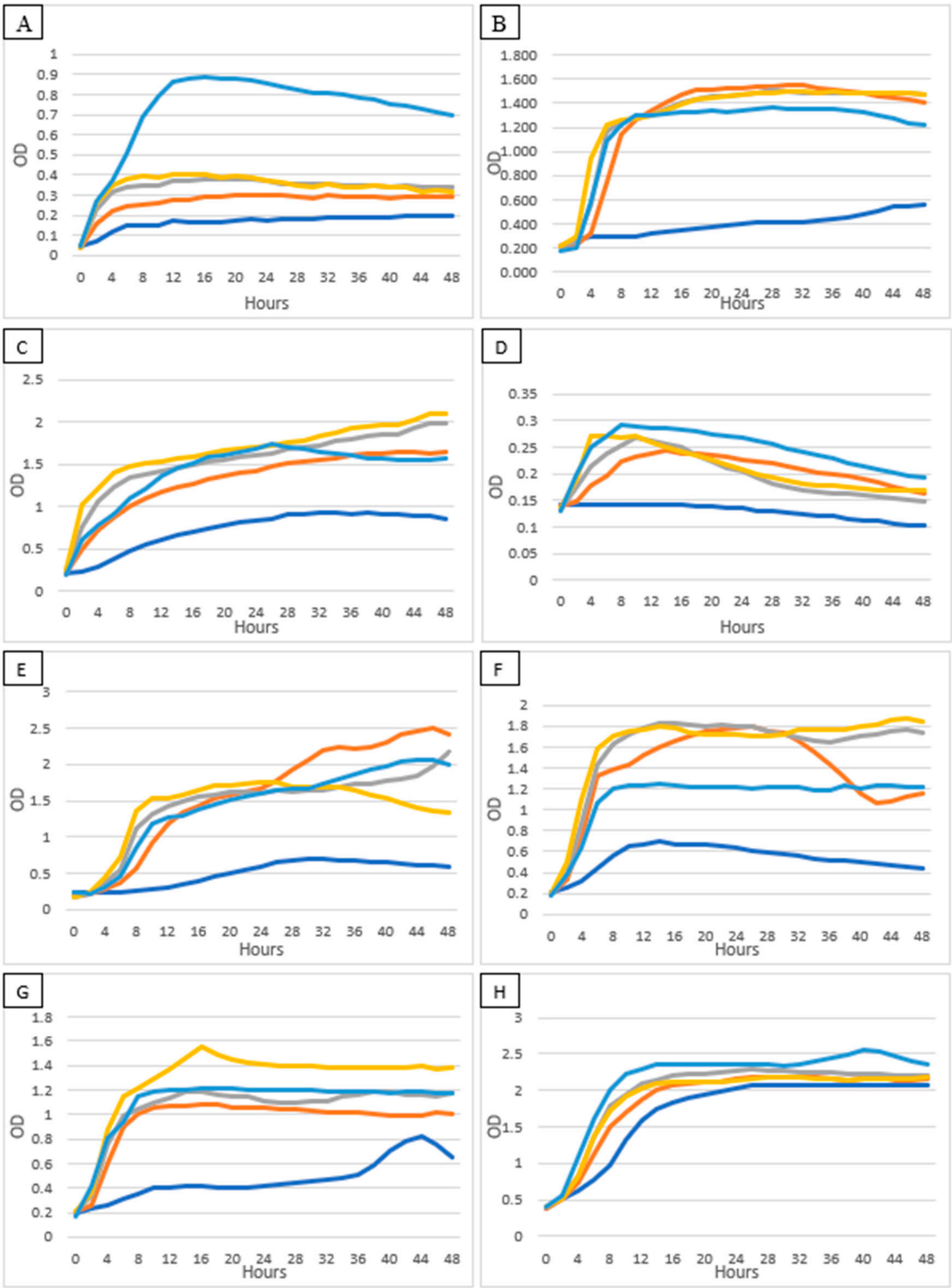

Pōhutukawa honey was tested with eight bacteria at four concentrations and a control. Corresponding graphs a) *E. coli* b) *B. subtilis* c) *S. typhimurium* d) *E. faecalis* e) *P. aeruginosa* f) *S. epidermidis* g) *S. aureus* h) *L. plantarum*. Corresponding concentration levels 25.00% 12.50% 6.25% 3.13% Positive control

Supplementary Figure S5

Broth Microdilution Assay growth curves with kānuka honey

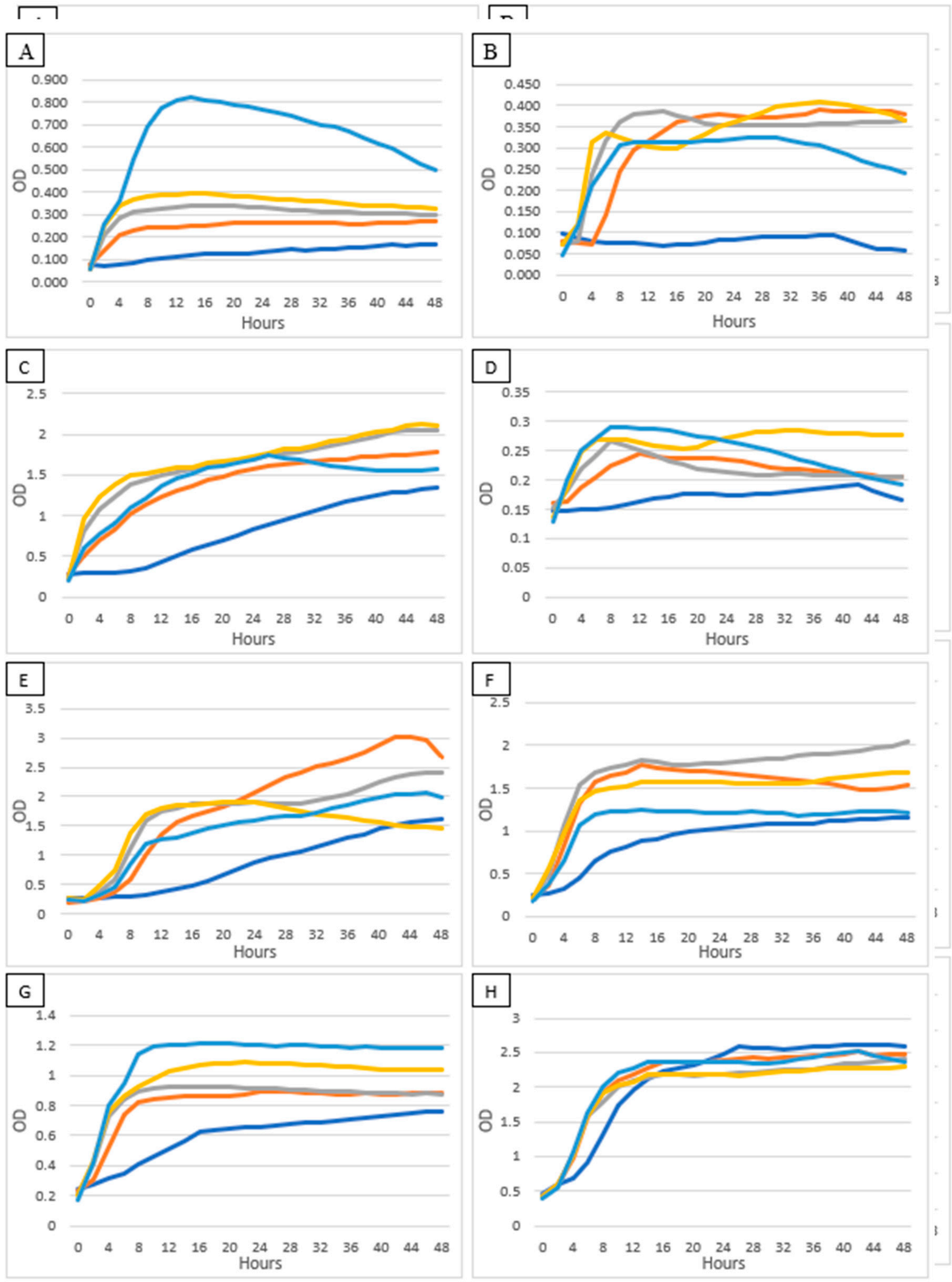

Kānuka honey was tested with eight bacteria at four concentrations and a control. Corresponding graphs a) *E. coli* b) *B. subtilis* c) *S. typhimurium* d) *E. faecalis* e) *P. aeruginosa* f) *S. epidermidis* g) *S. aureus* h) *L. plantarum*. Corresponding concentration levels 25.00% 12.50% 6.25% 3.13% Positive control

Supplementary Figure S6

Broth Microdilution Assay growth curves with kāmahi honey

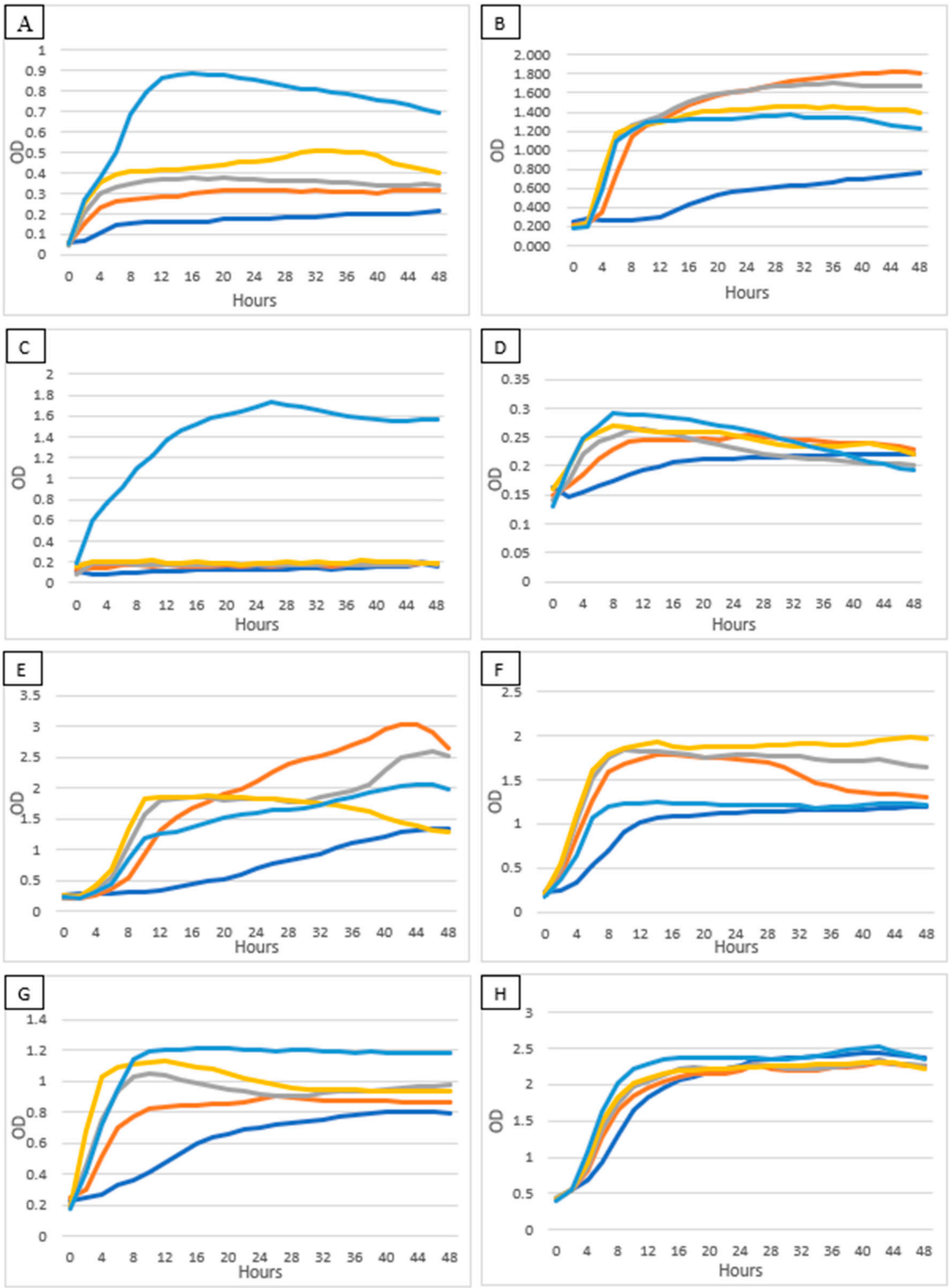

Kāmahi honey was tested with eight bacteria at four concentrations and a control. Corresponding graphs a) *E. coli* b) *B. subtilis* c) *S. typhimurium* d) *E. faecalis* e) *P. aeruginosa* f) *S. epidermidis* g) *S. aureus* h) *L. plantarum*. Corresponding concentration levels 25.00% 12.50% 6.25% 3.13% Positive control

Supplementary Figure S7

Broth Microdilution Assay growth curves with thyme honey

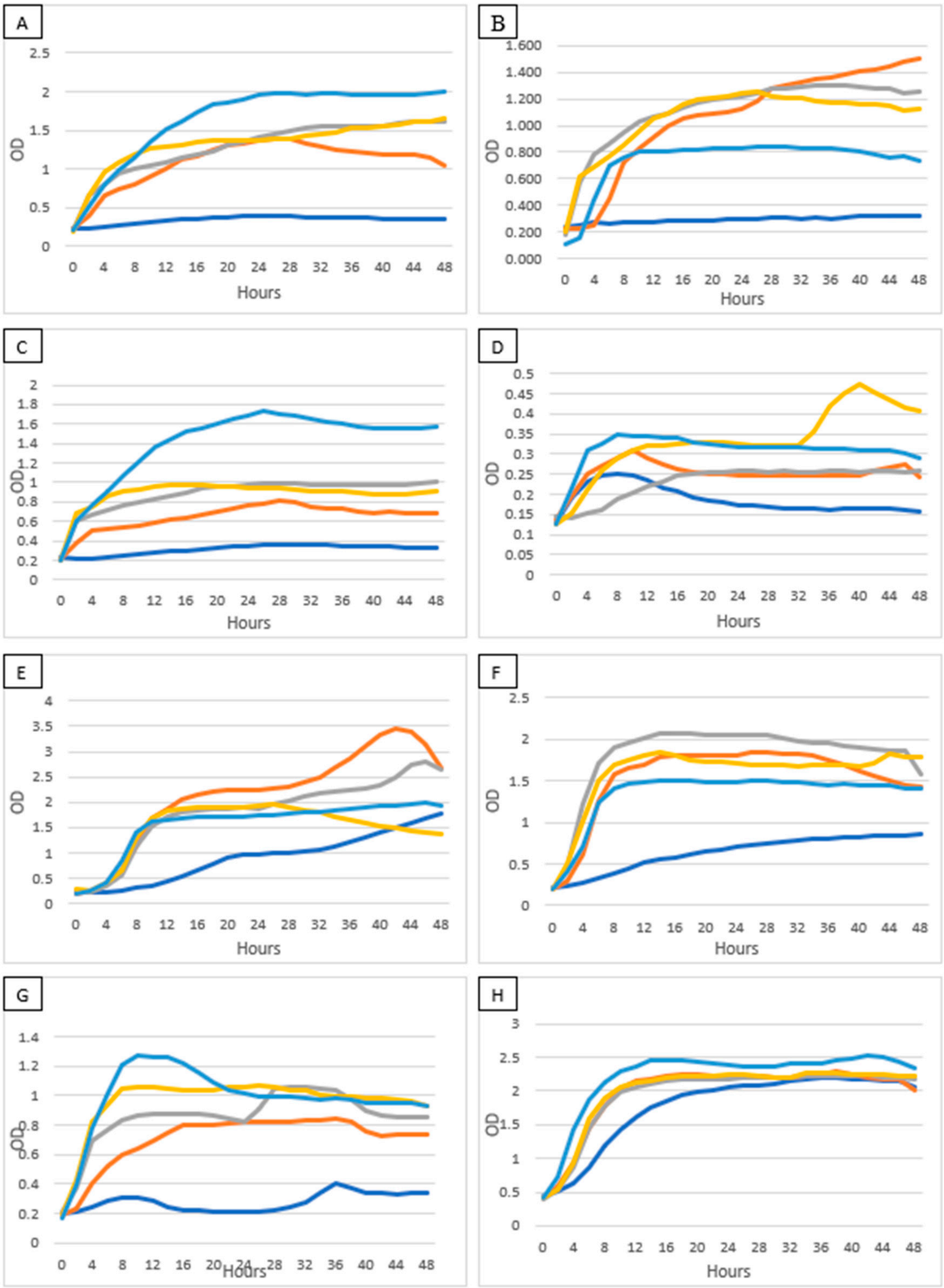

Thyme honey was tested with eight bacteria at four concentrations and a control. Corresponding graphs a) *E. coli* b) *B. subtilis* c) *S. typhimurium* d) *E. faecalis* e) *P. aeruginosa* f) *S. epidermidis* g) *S. aureus* h) *L. plantarum*. Corresponding concentration levels 25.00% 12.50% 6.25% 3.13% Positive control

Supplementary Figure S8

Broth Microdilution Assay growth curves with artificial honey

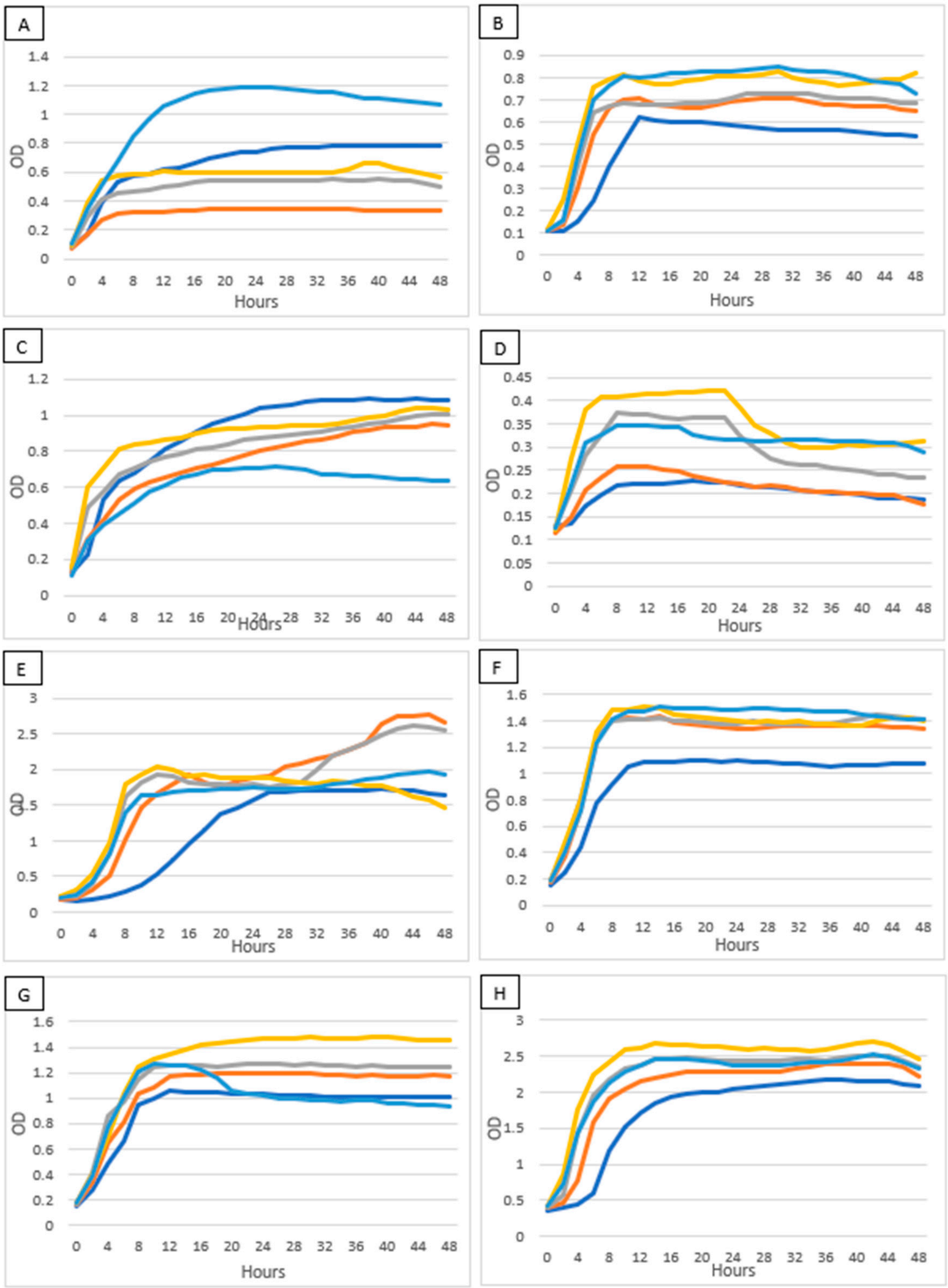

Artificial honey was tested with eight bacteria at four concentrations and a control. Corresponding graphs a) *E. coli* b) *B. subtilis* c) *S. typhimurium* d) *E. faecalis* e) *P. aeruginosa* f) *S. epidermidis* g) *S. aureus* h) *L. plantarum*. Corresponding concentration levels 25.00% 12.50% 6.25% 3.13% Positive control

Supplementary Figure S9a. AGP diffusion gel assay of honeys; from left to right Arabic controls of 1.25mg, 2.5mg, 5mg, Clover, Manuka, Honeydew and Pohutukawa..

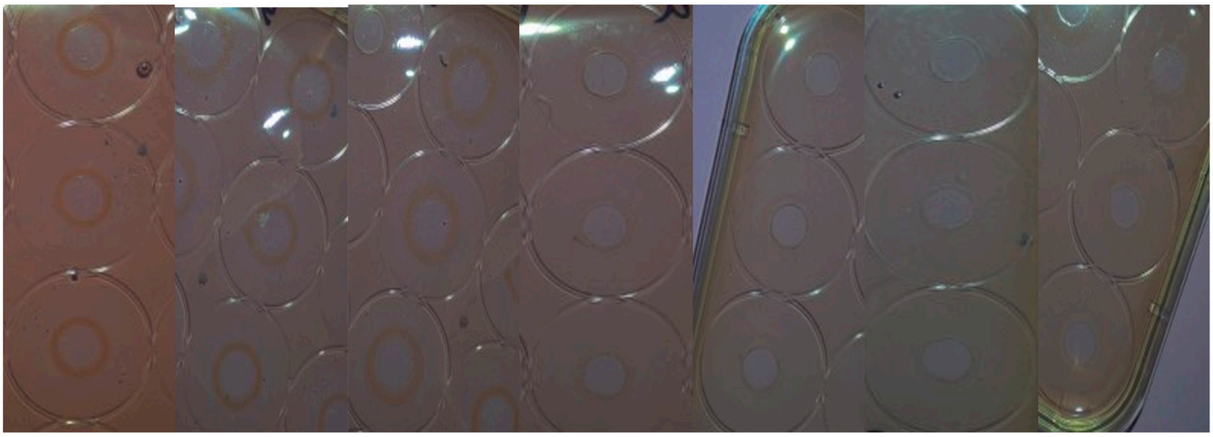

1.25mg/ml 2.5mg/m 5mg/ml Clover
Manuka Honeydew Pohutukawa

Supplementary Figure S9b. AGP diffusion gel assay of honeys; from left to right Arabic controls of 1.25mg, 2.5mg, 5mg, Kanuka, Rewarewa, Kamahi and Thyme

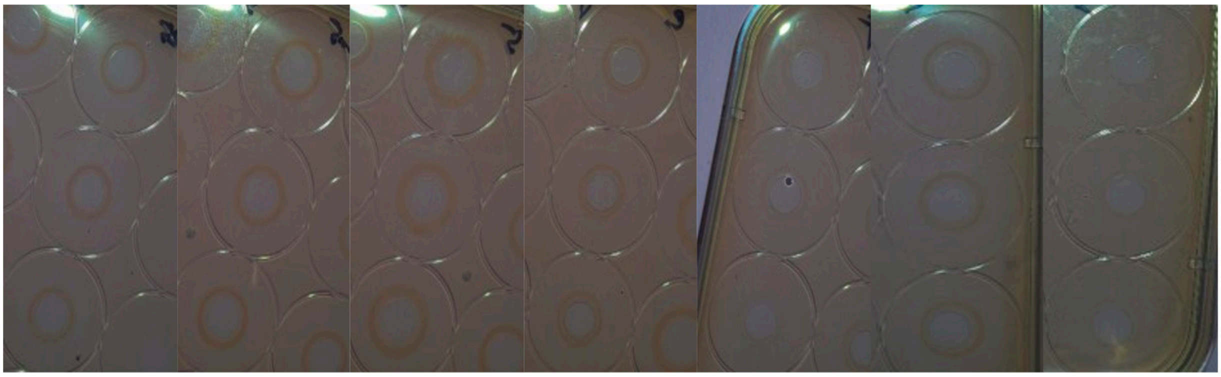

1.25mg/ml 2.5mg/m 5mg/ml Kanuka
Rewarewa Kamahi Thyme
